# Functionalization of Carbon Nanotubes with 5‑Phenylvaleryl, Pentadecanoyl, and Adipoyl Chloride: Synthesis and Spectroscopic Surface Analysis Toward Biomarker Sensing

**DOI:** 10.1021/acsomega.6c06619

**Published:** 2026-10-28

**Authors:** Ashley Galanti, Amin Trabelsi, Ryan Maynard, Maximo Reyes, Ramaraja Ramasamy, Mark Haidekker

**Affiliations:** † College of Engineering, 1355 University of Georgia, Athens, Georgia 30602, United States; ‡ College of Veterinary Medicine, University of Georgia, Athens, Georgia 30602, United States; § Ivester College of Health Sciences, 3461 Brenau University, Gainesville, Georgia 30501, United States

## Abstract

Carbon nanotubes (CNTs) were covalently modified with aromatic (5-phenylvaleryl, 5PC), long-chain aliphatic (pentadecanoyl, PDC), and oxygen-rich/carboxylated (adipoyl, ACC) chemistries to create a tunable surface interface for detecting volatile and semivolatile chemical biomarkers. Following oxidative activation, 3-aminopropyltriethoxysilane (APTES) amination, and acyl-chloride coupling, successful attachment and differentiation of linker classes were evaluated using X-ray photoelectron spectroscopy (XPS), ultraviolet photoelectron spectroscopy (UPS), and C KLL Auger analysis. Distinct spectroscopic fingerprints corresponding to aromatic, aliphatic, and oxygenated functionalities were resolved despite the intrinsically carbon-rich substrate. These orthogonal signatures provide a compact quality-control strategy for CNT functionalization and establish a framework for correlating surface chemistry with predicted analyte interactions, including aromatic interactions, nonpolar adsorption, and acid–base or hydrogen-bond pairing. This surface chemistry palette is intended to enable flexible CNT-based sensor arrays for noninvasive monitoring of clinically relevant biomarkers, including ketones associated with diabetes, nitrogen-containing species linked to chronic kidney disease, and volatile compounds implicated in preictal seizure states. Sensor-level performance was not evaluated in the present study and is the subject of ongoing device work.

## Graphical abstract

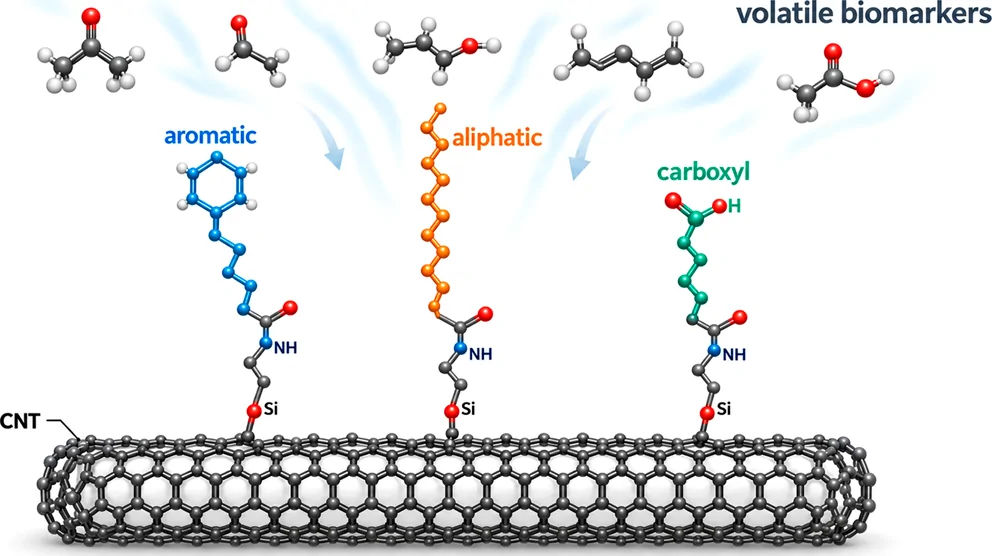

## Introduction

Real-time detection of chemical biomarkers is being explored as a noninvasive strategy for monitoring disease-relevant physiological states.^1–3^ Prior studies have shown that volatile and semivolatile compounds in breath, perspiration, and related biofluids can provide clinically useful information for conditions including diabetes, chronic kidney disease, and epilepsy.^1,3,5,6^ In particular, certain volatile organic compounds have been reported to change before seizure onset, suggesting potential utility for preictal monitoring.^1,7^ Because these biomarkers span diverse chemical classes, their detection may require sensor interfaces with different affinities for aromatic, nonpolar, and polar analytes.^2,8^ Carbon nanotubes (CNTs) are well suited for this purpose because their high surface area and conductive network structure enable sensitive transduction of interfacial adsorption events.^8–12^ Surface functionalization therefore provides a practical route to tuning CNT-analyte interactions and to developing cross-reactive sensor platforms for differential biomarker detection.^2,9,11,13^

While seizure prediction motivates the present work, the same sensing principles extend to a wider set of clinical conditions in which breath or skin headspace VOCs correlate with disease activity.^1,3,5^ In diabetes, elevations of acetone and related ketones accompany disordered glucose and fat metabolism, reflecting glycemic control;^4^ in chronic kidney disease, impaired nitrogen handling elevates ammonia and low-molecular-weight amines in breath.^5,6^ Infectious, hepatic, and inflammatory diseases have also been linked to reproducible VOC patterns that shift with disease state. Broadening the scope from a single neurologic indication to a generalizable VOC interface therefore increases the impact and translational value of the platform while preserving the original motivation of detecting preictal changes.^1,3,5^ In this expanded view, the same underlying transducer can be configured to target families of biomarkers relevant to seizures as well as to metabolic and renal disorders, enabling a modular approach to noninvasive monitoring.^2,8^

Here, the focus is placed on covalent functionalization of single-walled CNTs using three representative organic groups selected to emulate distinct classes of biomarker interactions: an aromatic phenyl moiety (5PC), a long-chain aliphatic group (PDC), and a carboxylic acid-terminated chain (ACC). These chemistries were chosen to span aromatic, nonpolar, and polar interaction modes, enabling differential affinity toward chemically diverse volatile biomarkers.^2,8,13^ In the context of seizure-related VOCs, and more broadly across metabolic and renal disease markers, this complementary surface chemistry palette supports discrimination among candidate analytes and facilitates the construction of modular sensor arrays without modification of the underlying transducer.^2,8^

Direct covalent functionalization of CNTs is challenging because pristine CNTs consist primarily of inert sp^2^ carbon.^2,14,15^ To enable attachment of molecules, reactive sites or functional handles must first be introduced, commonly through oxidative treatments that generate oxygen-containing surface groups.^16–18^ In this work, oxidative hydroxylation using potassium permanganate in sulfuric acid (KMnO_4_/H_2_SO_4_) was used to decorate CNT surfaces with oxygen-containing functionalities.^19–21^ Because acidic permanganate treatment can also introduce sidewall defects and shorten nanotubes at high oxidant loadings and long reaction times,^16,19,21^ a 1:1 (w/w) KMnO_4_:CNT ratio was used, and retention of the conjugated sp^2^ network was assessed indirectly from the UPS π band, the C 1s line shape, and the C KLL D-parameter (see Discussion). These oxygenated sites (e.g., −OH, −COOH) provide anchoring points for subsequent coupling chemistry.^9,15,16^

Surface modification of CNTs for chemical sensor interfaces has been reviewed extensively.^37–39^ Noncovalent strategies, including polymer wrapping, π-stacking of aromatic small molecules, and ionic-liquid or metal-nanoparticle decoration, preserve the intrinsic sp^2^ network and have been widely used to tune chemiresistor selectivity toward breath-relevant VOCs.^8,34,35,37^ Covalent strategies instead anchor receptors at defect or oxygenated sites, most commonly through carboxylic acid groups generated by acid oxidation and subsequently coupled via carbodiimide (EDC/NHS) activation, acyl chloride intermediates, or silane coupling agents such as APTES.^15,24,26,38,39^ Covalent attachment gives a more robust receptor layer than physisorption, at the cost of partial disruption of the conjugated network, and has been used to introduce amine, carboxyl, alkyl, and aromatic terminations for gas and vapor sensing.^13,37,38^ Cross-reactive arrays built from differently modified CNT sensors have been shown to discriminate breath-related VOC mixtures when combined with pattern recognition.^2,33,34^ However, most reported arrays combine chemically unrelated receptor types (e.g., polymers, ionic liquids, metal nanoparticles), so that differences between sensor elements arise from both the receptor chemistry and the attachment method. To our knowledge, a systematic series in which aromatic, aliphatic, and carboxyl terminations are installed through an identical covalent linker and verified side by side by surface spectroscopy has not been reported.

To install the target groups, a two-step approach was used. Oxidized CNTs were first aminated via grafting of 3-aminopropyltriethoxysilane (APTES) to introduce surface-bound primary amines.^22–25^ Acyl chlorides were then coupled to these amines to form amide linkages, yielding terminal aromatic (5PC), long-chain aliphatic (PDC), or carboxylic acid-terminated (ACC) functionalities.^24,26^ Side-by-side comparison with appropriate controls enabled assessment of coupling and spectroscopic discrimination of each functional group on the carbon-rich surface.

A suite of surface analytical techniques was used to characterize each stage of functionalization. Energy-dispersive X-ray spectroscopy provided initial elemental indication, while X-ray photoelectron spectroscopy served as the primary tool for verification of chemical bonding and quantification of elemental composition.^14,27^ Ultraviolet photoelectron spectroscopy was used to probe valence-band features that differentiate aromatic and aliphatic linkers, and C KLL Auger analysis provided a complementary indicator of carbon hybridization.^27–29^ Together, these orthogonal measurements provide a compact fingerprint of each functionalization and address the challenge of identifying targeted groups when carbon signals overlap on a carbonaceous substrate.^14,27^

Within this framework, the objective of this work is 2-fold. First, a reproducible synthesis and unambiguous surface characterization of 5PC-, PDC-, and ACC-modified CNTs is established using orthogonal spectroscopic techniques capable of differentiating aromatic, aliphatic, and oxygenated linkers on a carbon-rich background. Second, the resulting interaction palette is mapped to plausible analyte classes to inform the design of modular sensor arrays for noninvasive monitoring across multiple disease areas. By correlating linker chemistry with spectroscopic signature and predicted biomolecular interactions, this platform provides a chemically versatile CNT-based foundation that can be extended from preictal seizure volatiles to clinically relevant VOC panels associated with metabolic and renal disorders, without modification of the underlying transducer architecture. The novelty of this work lies in two aspects. First, aromatic, aliphatic, and carboxyl terminations are installed through a single, identical APTES–amide linkage, so that spectroscopic differences between samples can be attributed to the terminal group rather than to the attachment chemistry. Second, we show that, whereas core-level XPS alone cannot reliably discriminate these terminations on a carbon-rich substrate, the combination of UPS valence-band features and the C KLL D-parameter provides a distinguishing fingerprint for each. Device-level sensing measurements are outside the scope of the present study.

## Methods

### Materials

Semiconductor-enriched single-walled carbon nanotubes (s-SWCNTs; 95% semiconducting purity, thick film; SKU 1106) were obtained from NanoIntegris. Absolute ethanol (200 proof, for molecular biology; E7023) and *N*,*N*-dimethylformamide (DMF; biotech. Grade, ≥99.9%; 494,488; dried before use) were purchased from Sigma-Aldrich, as were sulfuric acid (H_2_SO_4_, 18 M), potassium permanganate (KMnO_4_), hydrochloric acid (HCl, 12 M), acetone, 3-aminopropyltriethoxysilane (APTES), thionyl chloride (SOCl_2_), and tetrahydrofuran (THF). 5-Phenylvaleric acid (>99.0%; P1284) and adipic acid (>99.0%; A0161) were purchased from TCI, and pentadecanoyl chloride (PDC, 98%; QH-9769) from Combi-Blocks. All reagents were used as received. Deionized (DI) water was used throughout. Glassware for acyl chloride synthesis and coupling was flame-dried, and reactions were handled under N_2_.

### Initial CNT Oxidation

The starting material was semiconductor-enriched single-walled carbon nanotubes (s-SWCNTs) in powder form (NanoIntegris). Prior to functionalization, the CNTs were oxidatively “activated” to introduce hydroxyl functional groups.^16,19,21^

### Oxidation (Hydroxylation) of CNTs

Approximately 5 mg of CNTs were dispersed in 5 mL of 5 M H_2_SO_4_ in a glass tube. Potassium permanganate (KMnO_4_) was added slowly to the acidic suspension, a ∼5 mg total, introduced in small aliquots of an 83.33 mg/mL aqueous stock solution. The mixture was subjected to ultrasonication in a thermostated bath (160 W, 40 kHz) at 70 °C for approximately 15 h. This oxidative treatment promotes the formation of oxygen-containing functional groups on the CNT surface;^19–21^ however, prolonged acidic permanganate treatment at elevated temperature can also introduce defects and shorten nanotubes.^16,19,21^ A 1:1 (w/w) KMnO_4_:CNT ratio was therefore used, and the integrity of the conjugated network after functionalization was evaluated indirectly (see Results and Discussion). Following oxidation, the mixture was allowed to cool and transferred to centrifuge tubes for extensive washing to remove residual reagents and byproducts. The oxidized CNT suspension was diluted with deionized (DI) water and ultracentrifuged (20,000 rpm, 15 °C, 15 min) to pellet the CNTs. The supernatant containing residual acid and manganese salts was decanted, and the pellet was redispersed in fresh DI water by vortexing and brief sonication. This washing procedure was repeated multiple times. After the DI water washes, a wash with 12 M HCl was performed to dissolve and remove residual MnO_2_ or manganese-containing species.^19,21^ Following the acid wash, the CNTs were subjected to additional DI water washing cycles until the supernatant was neutral and clear. In total, approximately 4–5 centrifugation cycles (water and acid) were performed. The final oxidized CNTs (hereafter “OH–CNTs”) were dried under vacuum at room temperature prior to further use.

To verify the introduction of oxygen-containing functional groups, a small sample of dried OH–CNTs was analyzed by energy-dispersive X-ray spectroscopy (EDX). Samples were deposited on a SiO_2_ substrate and analyzed at a low acceleration voltage (5 kV) to enhance surface sensitivity for light elements. EDX spectra were collected for both oxidized and unfunctionalized CNTs, and elemental compositions were quantified for comparison. The observed oxygen content was consistent with prior reports for similar oxidation treatments.^19–21^

### APTES Silanization of CNTs (Amine Functionalization)

Five mg of OH–CNT powder was dispersed in 5 mL of absolute ethanol in a glass vial or round-bottom flask and probe-sonicated (CGOLDENWALL touch-screen ultrasonic homogenizer, 650 W, 20 kHz, 40% amplitude) for approximately 5 min to ensure dispersion. APTES (10–15 μL) was then added to the CNT/ethanol suspension. The reaction mixture was refluxed at 70 °C for 4 h under ambient atmosphere using an air condenser.^22–25^ After completion of the reaction, the mixture was allowed to cool to room temperature prior to purification. The silanized CNTs (APTES-CNTs) were transferred to centrifuge tubes and washed sequentially with ethanol, acetone, and deionized (DI) water to remove unbound or weakly bound APTES and reaction byproducts. The reaction mixture was first diluted with ethanol and ultracentrifuged (20,000 rpm, 15 °C, 15 min) to pellet the CNTs, and the supernatant was discarded. The pellet was redispersed in fresh ethanol and centrifuged again under the same conditions; this ethanol wash was repeated twice. The CNTs were then washed with acetone for two cycles, followed by a final DI water wash. After the final wash, the APTES-CNTs were dried under vacuum at room temperature for approximately 12–24 h to yield a dry powder, and EDX spectra were collected from a small sample volume as described in the hydroxylation protocol.

### Synthesis of Acyl Chloride Functional Groups

To functionalize APTES-CNTs with phenyl, alkyl, and carboxyl end groups, acyl chloride coupling chemistry was employed.^24,26^ The carboxylic acid precursors 5-phenylvaleric acid and adipic acid were converted to their corresponding acyl chlorides using thionyl chloride under moisture-controlled conditions because of the hydrolytic sensitivity of acyl chlorides.^26^ For 5-phenylvaleric chloride (5PC), 0.89 g of 5-phenylvaleric acid (5 mmol) was added to a dry round-bottom flask under N_2_, followed by 1.45 mL SOCl_2_ (25 mmol) and a catalytic drop (∼0.1 mL) of dry DMF. The mixture was heated to ∼90 °C under N_2_ for 4 h, after which excess thionyl chloride was removed by two-stage distillation, first at ambient pressure and then under vacuum (<100 mTorr), to yield crude 5-phenylvaleric chloride as a pale yellow oil, which was used without further purification. For ACC, adipic acid (444 mg, ∼3 mmol) was combined with equimolar SOCl_2_ (∼360 mg, 0.22 mL, ∼3 mmol) in 10 mL dry THF with a catalytic drop of DMF and heated under N_2_ for 3 h. Because a 1:1 SOCl_2_:adipic acid ratio was used, the reaction likely produced predominantly adipoyl monochloride with some dichloride. Excess solvent was removed by two-stage distillation at 70 °C, first at ambient pressure and then under vacuum (<100 mTorr). PDC was purchased from Combi-Blocks (98% purity) and used as received.

### Covalent Attachment of Functional Groups to APTES-CNTs

This step was conducted separately for each functional group in dry, oxygen-free solvents. The general coupling reaction is shown in Scheme 1, and the resulting surface structures for each functional group are illustrated in Figures 1, 2, and 3.

**Scheme 1:**
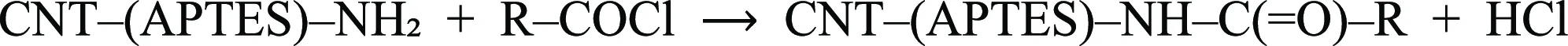
General amide coupling of acyl chlorides (R–COCl; R = 4-phenylbutyl for 5PC, tetradecyl for PDC, 4-carboxybutyl for ACC) to APTES-functionalized CNTs in anhydrous DMF under N_2_ at 100 °C for 24 h

**Figure 1:**
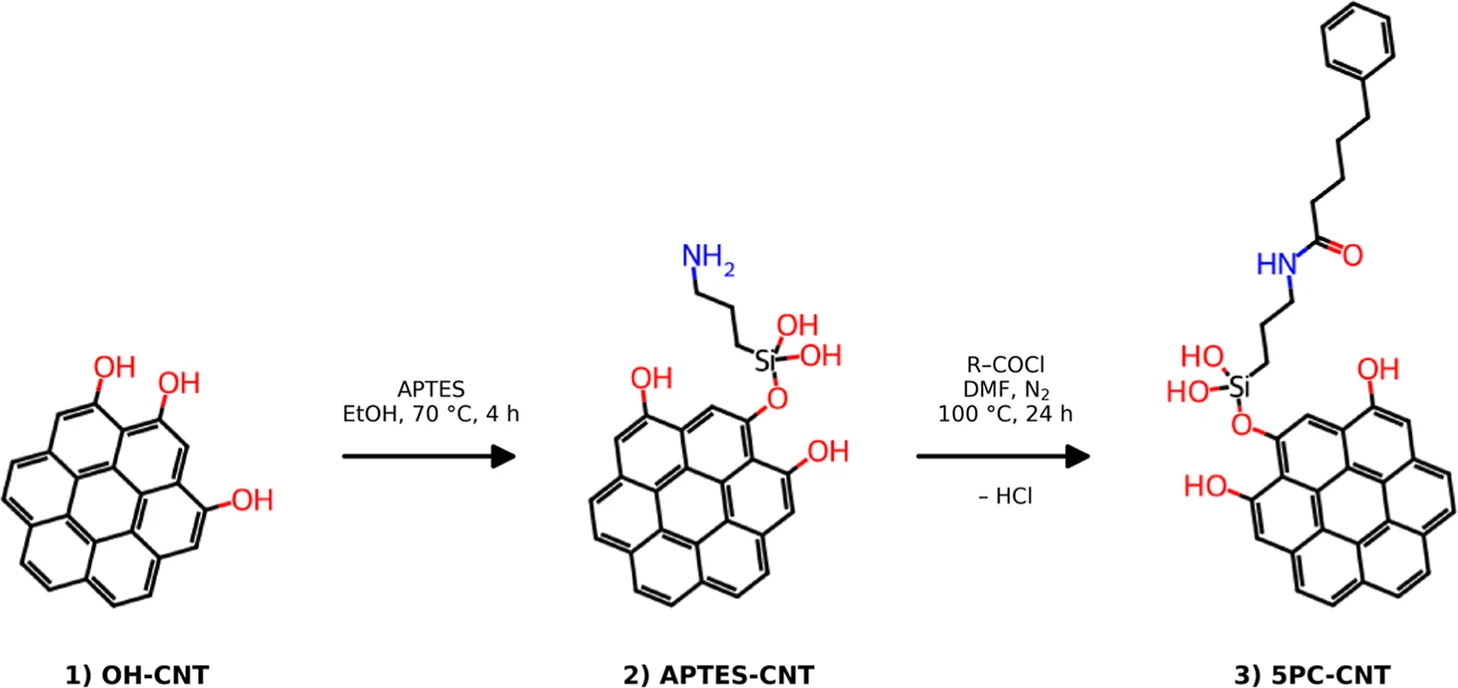
*3-Step functionalization of CNTs:* (1) OH–CNTs, (2) APTES-CNTs, (3) 5PC–CNTs. Structures drawn by the authors; the polyaromatic fragment represents a local region of the CNT sidewall, and the silane binding mode is idealized.

**Figure 2:**
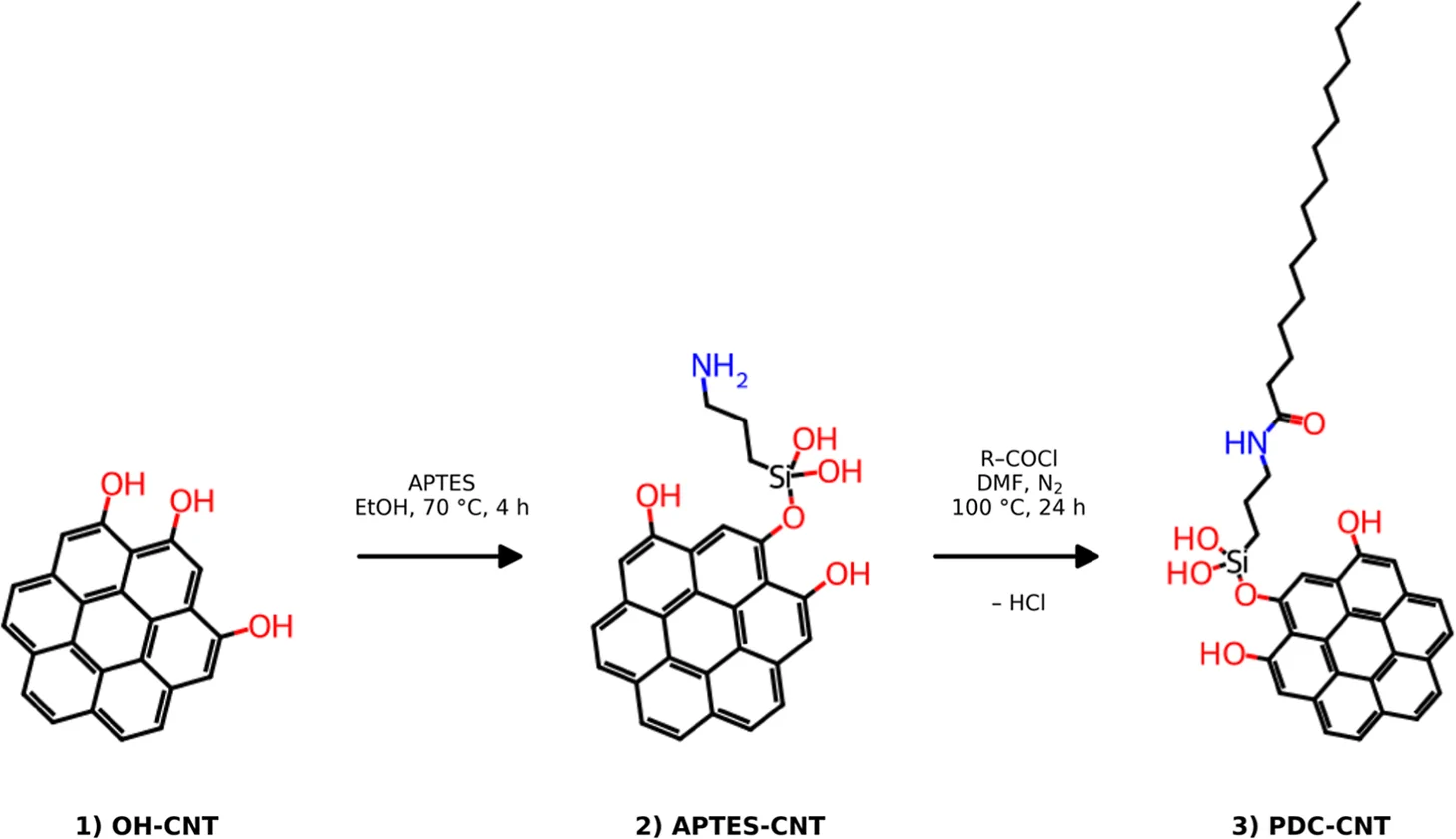
*3-Step functionalization of CNTs:* (1) OH–CNTs, (2) APTES–CNTs, (3) PDC–CNTs. Structures drawn by the authors; representation as in Figure 1.

**Figure 3:**
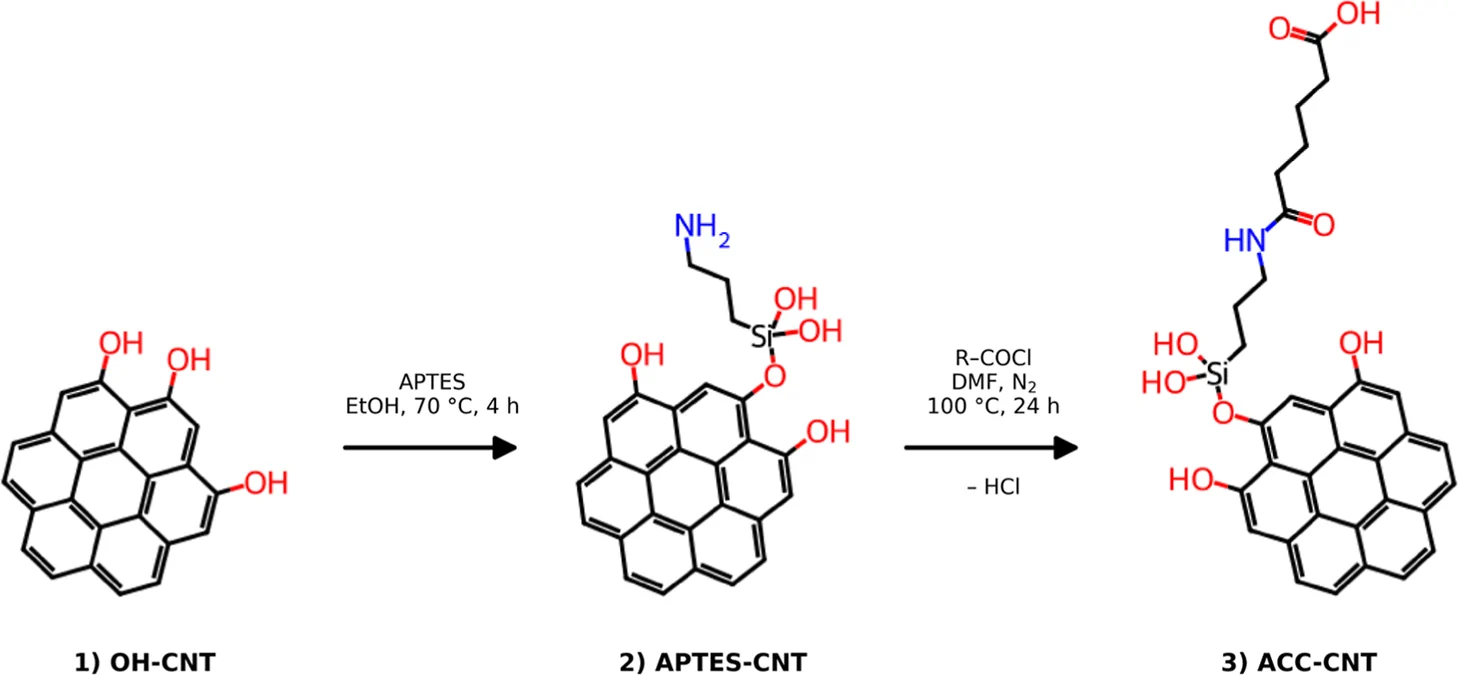
*3-Step functionalization of CNTs:* (1) OH–CNTs, (2) APTES-CNTs, (3) ACC–CNTs. Structures drawn by the authors; representation as in Figure 1.

Functionalization reactions were performed separately in dry, oxygen-free DMF. A stir bar and 1 mg dry APTES-CNTs were placed in a flame-dried round-bottom flask under N_2_. Anhydrous DMF (20 mL) was added, and the suspension was briefly sonicated before addition of ∼50 mg (∼50 μL) of the corresponding acyl chloride. The mixtures were stirred at 100 °C under N_2_ for 24 h, quenched with water, collected by centrifugation, washed once with water and three times with THF, and dried in a vacuum oven at 60 °C. For 5PC–CNT and PDC–CNT, 5-phenylvaleryl chloride and pentadecanoyl chloride were used directly, respectively. For ACC–CNT, adipoyl chloride was generated in situ as described above; after THF removal, the crude product was taken up in 50 mL dry DMF and reacted with APTES-CNTs under the same conditions, then washed and dried similarly. A small aliquot of each sample was reserved for analysis. The coupling reactions were performed at the ∼1 mg scale because this quantity exceeds what is required for both the intended application and the characterization. In the target sensor devices, each chip is prepared from ∼0.04 mg of CNTs, so that 1 mg supplies approximately 25 sensor chips, sufficient for a complete sensor array; in addition, each surface-sensitive analysis used here (XPS, UPS, Auger, EDX) consumes only microgram quantities. Working at this scale also limits consumption of the costly sorted s-SWCNT starting material. To limit physical losses, supernatants from every centrifugation cycle were retained and inspected for suspended CNTs before being discarded, a miniature stir bar was used to minimize adhesion of CNTs, and reactions were blanketed with N_2_ rather than evacuated to avoid aspirating the powder; after the oxidation and washing steps, approximately 5.0 mg of CNTs was recovered from 5.0 mg of starting material. Scale-up to 50–100 mg batches, which would also allow bulk characterization such as solid-state ^13^C NMR and thermogravimetric analysis, is planned for future device studies.

### Surface Analysis Techniques

After each stage of functionalization, CNT samples were characterized by EDX, XPS, UPS, and C KLL Auger analysis to evaluate surface composition and bonding.^14,27^ For EDX, CNT powders were dispersed in DMF and drop-cast onto gold-coated Si/SiO_2_ substrates. After solvent evaporation, spectra were collected using a scanning electron microscope equipped with an Oxford Aztec EDX system at 5 kV for 2–5 min per spot. Elemental weight and atomic percentages were obtained using standardless ZAF-corrected quantification. Because EDX is less reliable for light elements, the data were used comparatively to monitor the appearance of N and Si after APTES treatment and to check for residual Cl or S from incomplete washing.^14^ For XPS, CNT powders were mounted on indium foil or conductive carbon tape and analyzed using a Thermo Scientific Nexsa instrument with a monochromatic Al Kα source. Survey scans were collected first, followed by high-resolution scans of the C 1s, O 1s, N 1s, and Si 2p regions, with Cl 2p and S 2p acquired when relevant. Spectra were collected at a pass energy of 20 eV. Charge neutralization was applied as needed, and binding energies were referenced to the graphitic C–C peak of unfunctionalized CNTs at 284.5 eV.^14,30^ Data were analyzed using KherveFitting software with Shirley background subtraction and Gaussian peak fitting constrained by reported chemical shifts.^14,30,31^ The C 1s region was deconvoluted into sp^2^ carbon, sp^3^ carbon, C–O/C–N, and carbonyl contributions, while the N 1s region was used to distinguish amine and amide nitrogen after acylation.^14,23^ Atomic concentrations were calculated using sensitivity factors. UPS measurements were performed on the same sample types using a He I discharge lamp over a binding energy range of 0–20 eV relative to the Fermi level.^28,29^ Spectra were background-subtracted, normalized to equal total area, and compared with emphasis on the π region, σ-band edge, and higher-binding-energy shoulders associated with oxygen-containing groups.^27,28^ The 5PC–CNT, PDC–CNT, and ACC–CNT samples were measured in a single session to maintain consistent instrumental conditions; UPS was not collected for OH–CNT because of instrument time limitations. C KLL Auger spectra were obtained during XPS measurements and differentiated to determine the D-parameter from the energy separation between the main C 1s peak and the principal Auger feature.^27^ This parameter was used as a semiquantitative indicator of sp^2^/sp^3^ carbon character.^27^ The presence or absence of π–π* satellite structure was also evaluated to distinguish aromatic from more aliphatic surface modifications.^14,27^

Solid-state ^13^C magic-angle-spinning (MAS) NMR was considered but not performed. At the 1 mg reaction scale, the available sample is well below the quantity typically required for natural-abundance ^13^C MAS NMR of carbon nanomaterials (on the order of tens of milligrams); the grafted carbons represent only a small fraction of the total carbon; and the electrical conductivity of SWCNTs, together with any residual catalyst impurities, broadens resonances and complicates sample spinning. Surface-sensitive photoelectron spectroscopies, which probe the outermost few nanometers where the grafted groups reside, were therefore used as the primary characterization methods.

All measurements were carried out at room temperature. Data from the different techniques were cross-correlated to build a consistent understanding of the surface composition and bonding. The results are presented below by technique, together with a discussion of how each piece of evidence supports the successful functionalization of CNTs with the intended groups.

## Results

### Functionalization Process Observations

Presented below are the analytical results for each sample. For clarity, Table 1 summarizes the elemental composition (from EDX and XPS) of the samples at key stages.

**Table 1:** Surface Elemental Composition of CNT Samples by EDX and XPS^a^ ^b^

sample	EDX At.% C: O: N: Si: Cl: Au	XPS At.% C: O: N: Si: Cl: S	notes
**Pristine CNT**	100:0: 0:0: 0:0	–	As received from supplier
**OH–CNT (oxidized)**	77:13:0: 0:0: 10	–	EDX 10% Au artifacts (show up as F) from substrate
**APTES–CNT**	67:22:1: 10:0	–	New N, Si peaks indicate APTES; O from silane and residual −OH
**5PC–CNT**	45:3: 0:0: 52	88:7: 2:2: 0:1	Au from substrate; S from thionyl chloride; N and Si from APTES (fractional amounts seen by XPS)
**PDC–CNT**	41:3: 0:0: 56	90:7: 2:1: 0:0	Au from substrate; N and Si from APTES (fractional amounts seen by XPS)
**ACC–CNT**	37:3: 0:0: 60	88:7.5:2: 2:0.5:0	Au from substrate, highest fractional O composition across both methods; Cl from thionyl chloride; N and Si from APTES (fractional amounts seen by XPS)

a Atomic percentages, with XPS normalized to carbon = 100 for comparison of O/C ratios.

b EDX values are semiquantitative; XPS values are from survey scans calibrated to C 1s 284.8 eV. Hydrogen not detected in either. EDX samples were prepared on chip substrates containing Au and Si/SiO2 (deposited using solvent) while XPS samples prepared well sample holders (powder form). EDX is not used as the final 5PC v PDC v ACC distinction, just to indicate Cl groups gone after APTES and acyl chloride binding.

From Table 1, trends such as increased oxygen after oxidation and the appearance of N and Si after APTES (∼1 and ∼10% by EDX, suggesting silane attachment), and a slight increase in oxygen for the ACC–CNT, due to the introduced carboxyl, are observed. The F detected in OH–CNT by EDX (∼10%) is attributed to substrate-level artifacts. From Table 2, comparison of the carbon and oxygen compositions obtained from XPS analysis for 5PC, ACC, and PDC shows that ACC exhibits a slightly higher oxygen content. However, because XPS primarily probes core-level (K-shell) binding energies, it does not capture contributions from L-shell valence electrons, limiting its ability to further differentiate ACC from the other functionalizations.^27,29,31^

**Table 2:** XPS Data Normalized to Carbon for Oxygen Composition Comparison

	carbon (C 1s)	oxygen (O 1s)
5PC	91.75%	8.25%
PDC	92.54%	7.46%
ACC	91.57%	8.43%

Next, detail of the XPS and UPS spectra, which provide direct evidence of the bonding environment for each functional group.

### XPS Survey and Elemental Analysis

The XPS survey spectra of the functionalized CNTs (Figure 4) show peaks corresponding to C 1s (∼284 eV) as the dominant feature for all samples, as expected. In addition, O 1s (∼532 eV) is present in all oxidized/functionalized samples. The APTES-containing samples exhibit an N 1s peak at ∼400 eV and a Si 2p peak at ∼102 eV.^14,22,23^ These indicate the presence of the silane coupling layer. A very small S 2p signal (∼168 eV) and Cl 2p (∼200 eV) were detectable in some survey scans, especially for the ACC sample, which are attributed to trace residues of sulfuric acid or thionyl chloride byproducts. In general, the survey spectra establish that relative to the starting CNT material (which would show only C and minor O), the functionalized CNTs contain significant O, as well as N and Si from APTES.^14,23^

**Figure 4:**
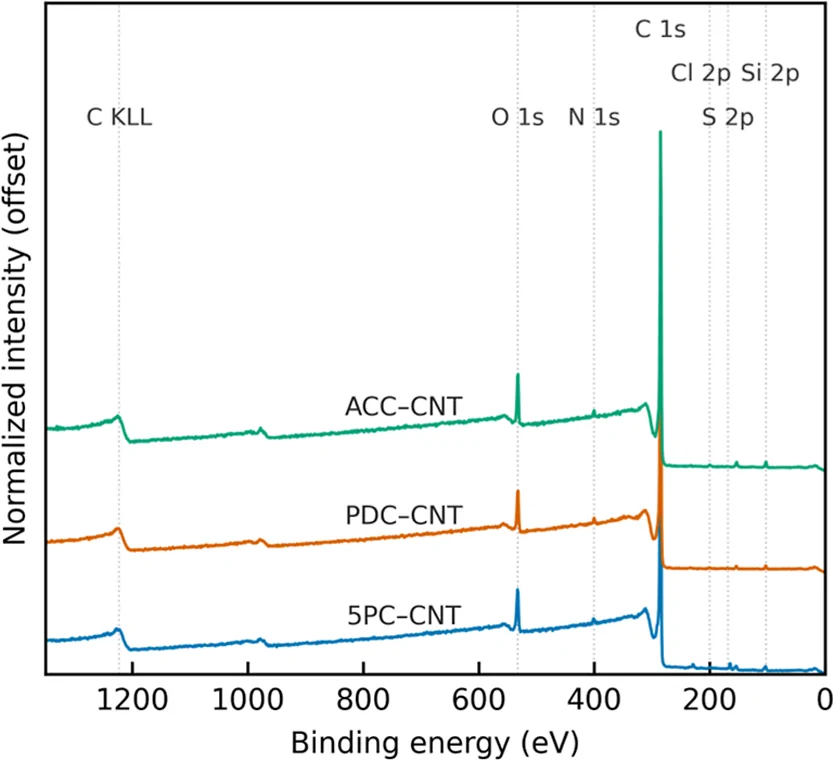
*XPS survey of functionalized CNTs.* Wide-range XPS survey spectra (normalized to the C 1s maximum and offset vertically) for 5PC–CNT (blue), PDC–CNT (orange), and ACC–CNT (green), showing the presence of O, N, and Si in all functionalized samples. The C 1s peak at ∼284.5 eV dominates. Dotted lines mark the C KLL, O 1s, N 1s (∼400 eV), Cl 2p, S 2p, and Si 2p (∼102 eV) positions.

Turning to the high-resolution **C 1s spectra** (Figures 5 and 6), the detailed peak shapes for each sample, as these are key to identifying functional groups. For clarity, Figure 5a overlays the C 1s XPS of the three differently functionalized CNTs, after subtraction of a Shirley background, and Figure 5b–d show the deconvolution for 5PC–CNT, PDC–CNT, and ACC–CNT, respectively. All spectra are calibrated so that the major graphitic C 1s peak (sp^2^ C–C) is at 284.5 eV.

**Figure 5:**
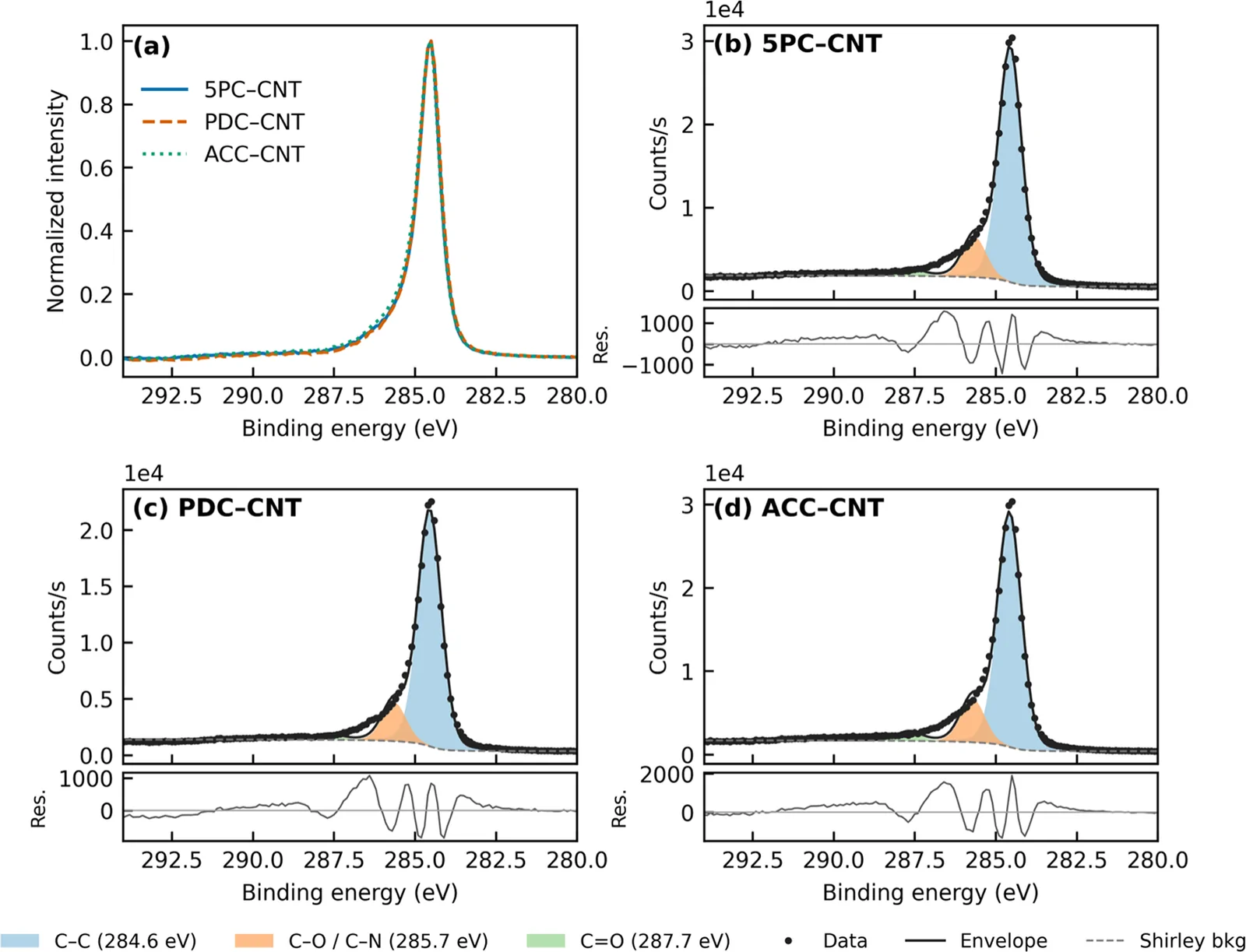
*High-resolution XPS C 1s spectra of functionalized CNTs with peak deconvolution.* (a) Overlaid C 1s spectra for 5PC–CNT (blue, solid), PDC–CNT (orange, dashed), and ACC–CNT (green, dotted), all normalized to the same height for the main peak. The C 1s region appears essentially identical across all samples, since the CNT backbone and carbon-containing functional groups produce overlapping signals at this excitation energy (K-shell); therefore, the spectrum is not diagnostic of functional group chemistry (L-shell) and is primarily useful for overall composition analysis. (b) Deconvolution of 5PC–CNT C 1s: major peaks at ∼284.5 eV (aromatic C–C), a smaller peak at ∼285.7 eV (C–O), and a clear amide C=O at ∼287.65 eV. (c) PDC–CNT C 1s: a dominant ∼284.5 eV sp^3^ C–C peak (from long alkyl), a still-present ∼285.6 eV sp^2^ C (from CNT backbone), C–O around 285.7 eV, and amide C=O ∼287.65 eV. The spectra composition mirror that of 5PC due to identical C with varying bonding orientations. (d) ACC–CNT C 1s: besides ∼284.5 eV (sp^2^) and ∼285.7 eV (C–O), there is a slightly higher peak at ∼287.65 eV (amide C=O) compared to 5PC and PDC, uniquely identifying the adipoyl functional group. Peak areas are consistent with one amide and one carboxyl per attached group. All spectra were charge-corrected to C–C at 284.5 eV.

**Figure 6:**
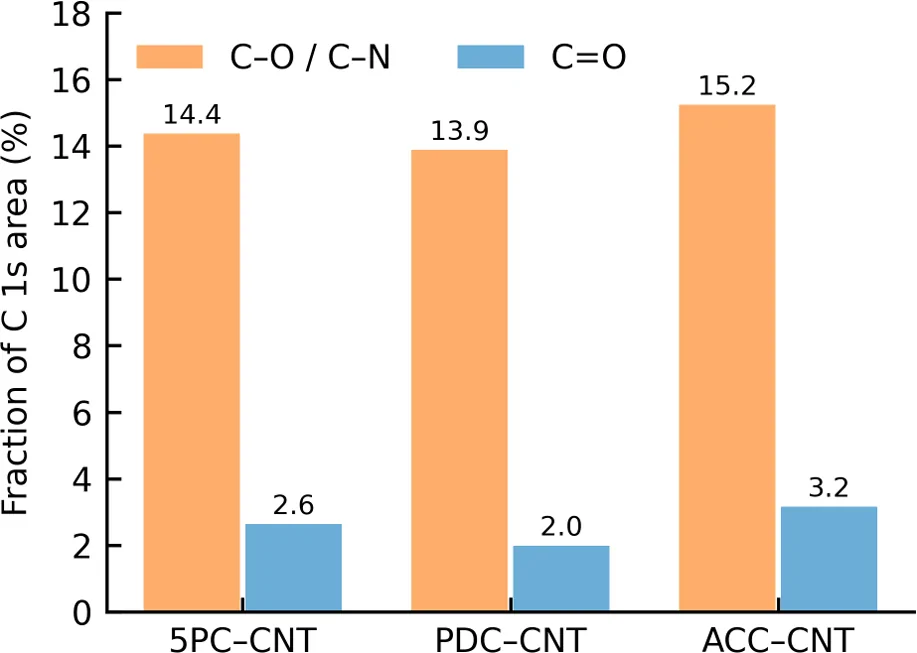
Relative atomic percentages of C–O/C–N and C=O components (from Tables 3–5) are shown for each sample. While all samples are dominated by the CNT C–C contribution, ACC–CNT exhibits a slightly higher fraction of oxygenated carbon species (C–O and C=O) compared to PDC–CNT and 5PC–CNT. This trend is consistent with the presence of both amide and additional carboxyl functionality in the adipic-derived linker, in agreement with the O 1s analysis.

Fitting: Shirley background; Gaussian–Lorentzian line shapes (15% Lorentzian) with a common fwhm for all components; constraints B = A + 1.2 ± 0.1 eV and C = A + 3.5 ± 0.4 eV. Conc. = component area as a percentage of the total fitted C 1s area; A/A = area relative to peak A; Split = position relative to peak A.

In 5PC–CNT (Figure 5b and Table 3), the C 1s spectrum can be fit with three main components: ∼284.5 eV corresponding to C–C from the CNT backbone and aliphatic segments, ∼285.7 eV attributed to C–O and/or C–N contributions from the linker chemistry, and ∼287.65 eV consistent with carbonyl (C=O) species. The appearance of the C=O peak indicates incorporation of oxygen-containing groups, while the dominant C–C signal reflects the underlying CNT structure. Thus, the C 1s deconvolution is consistent with the expected functionalization, though at this resolution it primarily serves to verify relative composition rather than distinguish specific bonding environments Figure 6.

**Table 3:** *5PC Deconvolution Data* ^a^

Peak	Assignment	Position (eV)	Height (CPS)	fwhm (eV)	Area (CPS·eV)	Conc. (%)	A/A (%)	Split (eV)
A	C–C	284.56	28,225	0.85	27,156	**83.0**	100.0	0.00
B	C–O/C–N	285.66	4889	0.85	4704	**14.4**	17.3	1.10
C	C = O	287.66	898	0.85	864	**2.6**	3.2	3.10

a C 1s peak-fit parameters for 5PC–CNT. Conc. = relative concentration of each component (% of total C 1s area); these values (bold) are the C–O/C–N and C=O fractions plotted in Figure 6.

Fitting: Shirley background; Gaussian–Lorentzian line shapes (15% Lorentzian) with a common fwhm for all components; constraints B = A + 1.2 ± 0.1 eV and C = A + 3.5 ± 0.4 eV. Conc. = component area as a percentage of the total fitted C 1s area; A/A = area relative to peak A; Split = position relative to peak A.

For PDC–CNT (Figure 5c and Table 4), the C 1s spectrum is also dominated by a strong C–C peak at ∼284.5 eV, with additional contributions at ∼285.7 eV (C–O/N species from the linker environment) and ∼287.65 eV (amide C=O from the pentadecanoyl linkage). The overall line shape is very similar to 5PC–CNT, consistent with the CNT backbone overwhelming any subtle functional group differences. As hypothesized, both 5PC and PDC yield nearly indistinguishable C 1s features in the K-shell, since the carbon framework and linker-derived carbons all overlap in this energy range. Any finer distinctions between aromatic versus aliphatic carbons would be more clearly resolved in the valence (L-shell) region by UPS rather than in these C 1s spectra.

**Table 4:** PDC Deconvolution Data^a^

peak	assignment	position (eV)	height (CPS)	fwhm (eV)	area (CPS·eV)	conc. (%)	A/A (%)	split (eV)
A	C–C	284.55	21,026	0.86	20,579	**84.1**	100.0	0.00
B	C–O/C–N	285.65	3471	0.86	3397	**13.9**	16.5	1.10
C	C=O	287.65	497	0.86	486	**2.0**	2.4	3.10

a C 1s peak-fit parameters for PDC–CNT; columns and bold values as in Table 3.

Fitting: Shirley background; Gaussian–Lorentzian line shapes (15% Lorentzian) with a common fwhm for all components; constraints B = A + 1.2 ± 0.1 eV and C = A + 3.5 ± 0.4 eV. Conc. = component area as a percentage of the total fitted C 1s area; A/A = area relative to peak A; Split = position relative to peak A.

For ACC–CNT (Figure 5d and Table 5), the C 1s spectrum again shows the dominant C–C peak at ∼284.8 eV, with additional contributions at ∼285.7 eV (C–O/N) and ∼287.66 eV (C=O). Unlike 5PC and PDC, however, ACC–CNT exhibits a more pronounced high-binding-energy C=O component, consistent with the presence of both amide and carboxyl carbons from the adipic fragment. This is in line with the survey composition analysis, which showed a slightly higher oxygen content in ACC–CNT relative to the other two samples. Although the difference is small, it is sufficient to distinguish ACC–CNT: its spectrum reflects not only the CNT backbone and amide linkage, but also the extra carboxyl functionality unique to this linker, as further shown in Figure 3. Thus, while the overall C 1s line shapes remain dominated by the CNT structure, ACC–CNT can be differentiated from 5PC and PDC by its relatively higher concentration of oxygenated carbon species Figure 6.

**Table 5:** ACC Deconvolution Data^a^

peak	assignment	position (eV)	height (CPS)	fwhm (eV)	Area (CPS·eV)	Conc. (%)	A/A (%)	split (eV)
A	C–C	284.58	28,124	0.87	27,946	**81.6**	100.0	0.00
B	C–O/C–N	285.71	5255	0.87	5221	**15.2**	18.7	1.13
C	C=O	287.68	1089	0.87	1081	**3.2**	3.9	3.10

a C 1s peak-fit parameters for ACC–CNT; columns and bold values as in Table 3.

Figure 7 highlights the O 1s region for the three final functionalized samples. While slight differences in peak position can be observed across 5PC–CNT, PDC–CNT, and ACC–CNT, after normalization it becomes clear that the dominant contribution arises from the underlying APTES/OH–CNT oxygen environment. As hypothesized, the oxygen signals from the attached functional groups are effectively overshadowed, resulting in nearly identical O 1s line shapes once normalized.^14,16^ This indicates that the O 1s feature primarily reflects the K-shell response of surface siloxane and hydroxyl oxygen, rather than providing a distinct fingerprint for each linker group. However, survey composition analysis reveals that the overall O atomic % remains slightly higher for ACC–CNT compared to 5PC–CNT and PDC–CNT, even after normalization to carbon (Table 2 and Figure 3). This difference is consistent with the extra oxygen present in the adipic-derived group and qualitatively supports its incorporation, even though its specific contribution is masked in the O 1s peak shape. Together, these results indicate successful functionalization across all samples, with ACC–CNT distinguished by its higher overall oxygen content despite the overlapping O 1s spectra.

**Figure 7:**
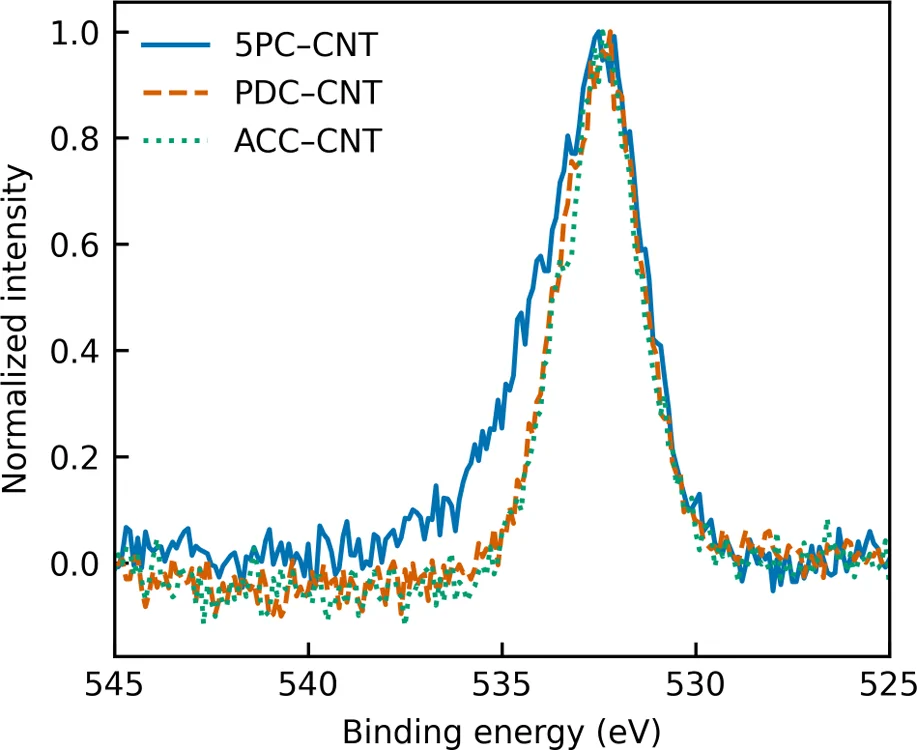
High-resolution XPS O 1s spectra of functionalized CNTs (Shirley background subtracted; normalized to the peak maximum): 5PC–CNT (blue, solid), PDC–CNT (orange, dashed), and ACC–CNT (green, dotted). Although slight peak position differences were observed, normalization reveals that the spectra are dominated by the APTES/OH–CNT oxygen environment, resulting in nearly identical line shapes. As hypothesized, oxygen signals from the functional groups are overshadowed in the O 1s region, making this feature primarily reflective of surface siloxane and hydroxyl contributions. Nevertheless, survey composition analysis indicates that ACC–CNT contains a slightly higher overall O content than 5PC–CNT and PDC–CNT, consistent with the additional oxygen from the adipic-derived linker, further differentiated through UPS analysis.

In summary, the XPS data indicate each functional group attachment: the presence of an aromatic shakeup for 5PC–CNT, high sp^3^ content for PDC–CNT, and a distinct carboxyl peak for ACC–CNT. All functionalized samples share the common signatures of the APTES linker (N 1s, Si 2p, amide C=O), verifying that the functionalization route, via APTES amine, was successful. The challenge of overlapping carbon signals is evident as the CNT’s own C 1s inevitably overlaps with new peaks, yet by careful analysis, these subtle differences could be delineated Figure 8.

**Figure 8:**
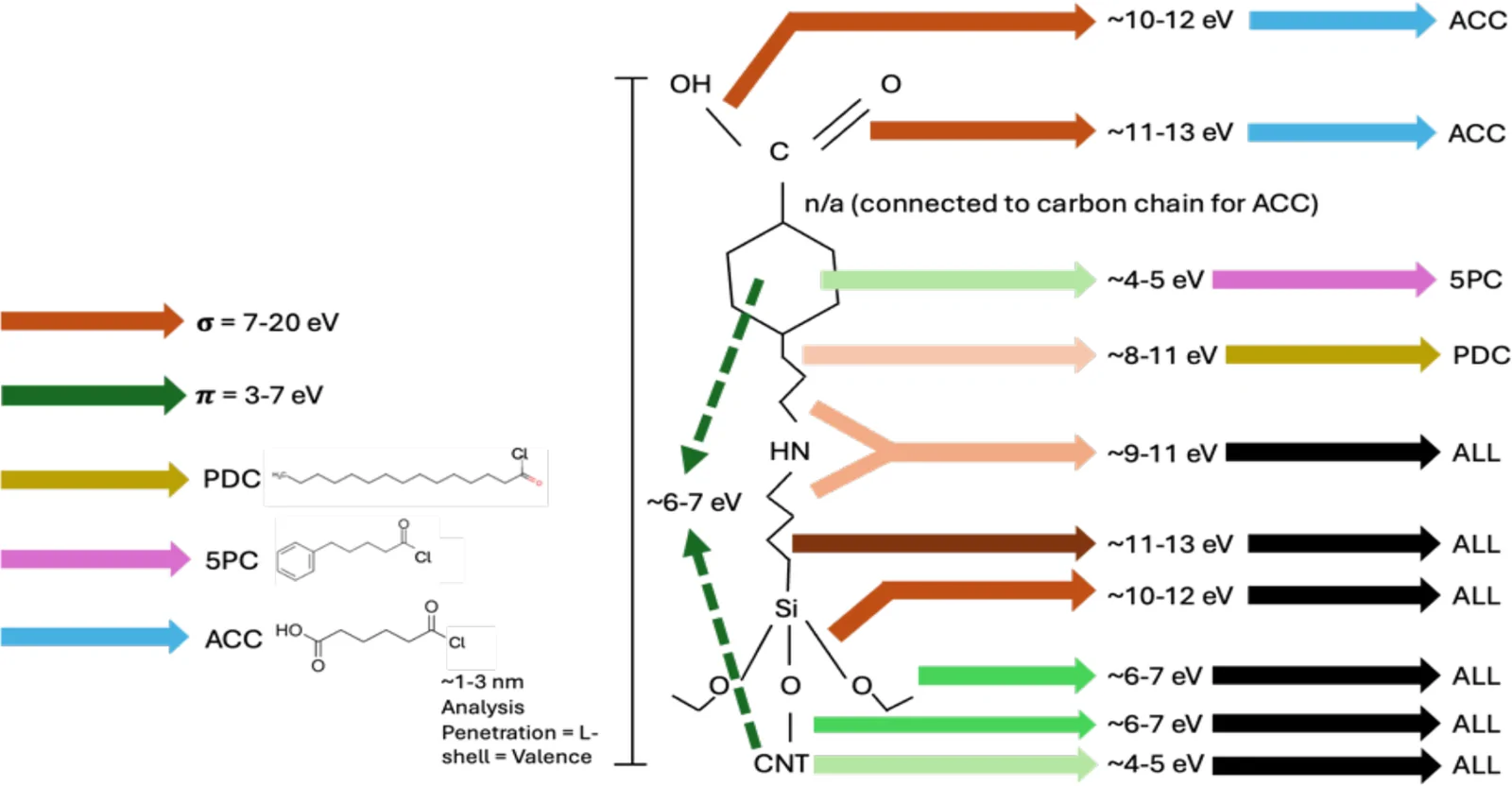
Schematic summary of UPS orbital assignments and functional group contributions for CNT functionalization. The table (left) lists main orbital contributions and their expected UPS binding energy ranges (He I, 21.22 eV). The schematic (right) maps these contributions to the CNT backbone and each functional group (5PC, PDC, ACC), highlighting the characteristic regions: π states (3–7 eV) from CNT and phenyl rings, σ states (7–20 eV) from aliphatic C–C and C–O/N bonds, and oxygen lone-pair features (∼11–13 eV) unique to ACC. Colored arrows correspond to functional groups: 5PC (pink), PDC (yellow), and ACC (blue), showing how each linker perturbs the CNT valence structure. This conceptual diagram integrates spectral assignments with chemical structure, providing a roadmap for interpreting UPS fingerprints of aromatic, aliphatic, and oxygenated modifications. It must be noted that the figure structure combines 5PC, PDC, and ACC as surface groups for analysis purposes and they are not connected on the actual sample.

### UPS Valence Band Analysis

Ultraviolet photoelectron spectra (He I, 21.22 eV) measurements provided complementary evidence of the changing electronic structure of the CNT surfaces upon functionalization.^27–29^ Figure 9a shows the full UPS valence spectra (0–20 eV binding energy) for a reference CNT sample and the three functionalized samples. All spectra were normalized to the total area under the curve for comparison. In our measurements, even the functionalized samples retain some features of the parent CNT electronic structure, but differences are observable.

**Figure 9:**
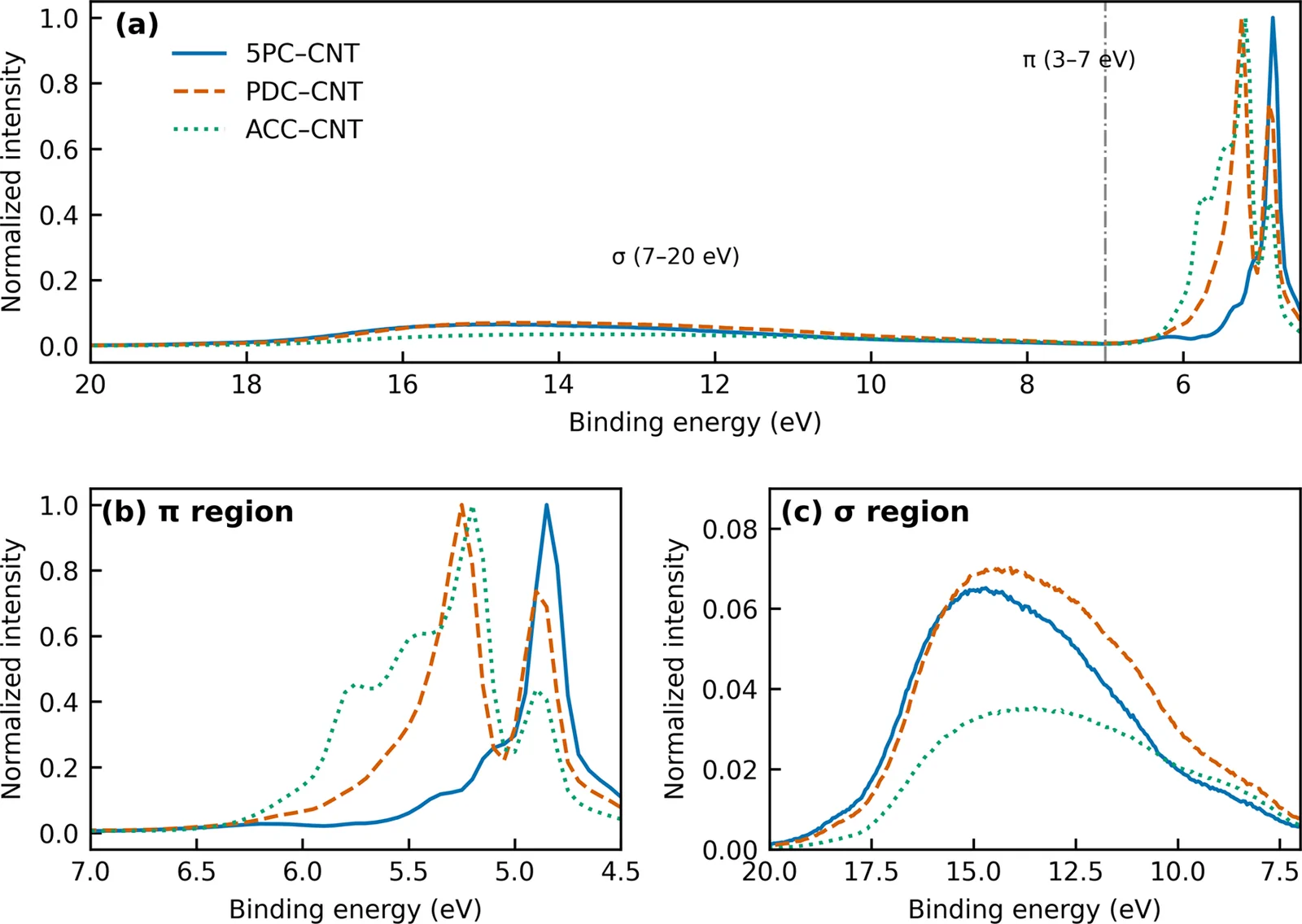
Ultraviolet photoelectron spectra (He I UPS, 3–20 eV binding energy) for functionalized CNTs: 5PC–CNT (blue, solid), PDC–CNT (orange, dashed), and ACC–CNT (green, dotted); spectra normalized to the π-band maximum. The valence band is divided into two regions: the π region (3–7 eV), dominated by emissions from sp^2^ (aromatic) carbon networks, and the σ region (7–20 eV), arising from sp^3^ C–C and C–X (X = heteroatom) bonds. **Panel (a)** shows the full UPS survey spectra, where all samples exhibit a prominent low-BE π band (peaks around 4–5 eV) and a broad higher-BE σ band (a hump centered near ∼14–16 eV). **Panel (b)** expands the 3–7 eV range to highlight differences in the π-band details. 5PC–CNT has the most intense π peak at the lowest binding energy (∼4.8 eV), attributable to its surface phenyl groups, with a shoulder near 5.2 eV from the underlying CNT π states. PDC–CNT shows a main π peak at ∼4.9 eV (from the CNT’s graphitic π orbitals) and a smaller π feature at ∼5.1 eV; the absence of aromatic functional groups in PDC–CNT means its π features derive purely from the nanotube. ACC–CNT displays a broadened π region with multiple peaks (∼4.9, 5.3, 5.5, 5.8 eV) due to the influence of its carboxylic linker–the electron-withdrawing C=O and −COOH groups split and shift the CNT’s π-band, introducing new occupied states at higher binding energies. The dash-dotted line at 7 eV marks the division between π and σ regions. In the σ region (7–20 eV), shown in **Panel (c)**, PDC–CNT produces the largest valence band hump (extending from ∼9 eV up to ∼16 eV) because of its long alkyl chains (rich in C–C σ bonds). 5PC–CNT shows a sizable but comparatively smaller σ band (due to a shorter linker and partial aromatic character), while ACC–CNT has the weakest σ intensity, consistent with its minimal sp^3^ carbon content. These UPS results illustrate how functional groups alter the CNT’s valence electronic structure: adding aromatic rings (5PC) intensifies π states, long aliphatic chains (PDC) amplify the σ band, and oxygen-containing linkers (ACC) introduce higher-BE π components and unique O 2p signatures in the valence band.

UPS of the functionalized CNT samples reveal distinct π-band (3–7 eV) and σ-band (7–20 eV) features, with clear differences attributable to each functional group.^27–29^ All three samples exhibit a prominent low-binding-energy π peak arising from the graphitic sp^2^ network of the CNTs, alongside broader high-binding-energy σ intensity from sp^3^ and heteroatom bonds.^27,28^ However, the position and intensity of these features vary markedly among 5PC–CNT, PDC–CNT, and ACC–CNT, reflecting differences in aromatic content, aliphatic (sp^3^) fraction, and oxygen functionalization.^27^

In the low-binding-energy π region (3–7 eV), shown in Figure 9b, each sample exhibits at least one peak arising from the delocalized π orbitals of the CNTs, although the surface functional groups clearly modulate these features.

5PC–CNT, which is functionalized with a phenyl group, displays the most intense and lowest-binding-energy π peak at approximately 4.8 eV. This peak is stronger and shifted to slightly lower binding energy than the corresponding CNT-only π feature, indicating a contribution from the highest occupied molecular orbital of the attached benzene ring.^27^ The phenyl group introduces additional aromatic π electrons at the surface, thereby enhancing the aromatic π signal.^27^ A shoulder is also observed at approximately 5.2 eV, which is attributed to the underlying π-band contribution from the CNT framework.^27^ The dominance of the 4.8 eV peak therefore highlights the aromatic character of 5PC–CNT, as the π states of the benzene group superimpose with and partially dominate the intrinsic π states of the nanotube surface.^27^

PDC–CNT, which is functionalized with a long alkyl chain, shows a π peak at approximately 4.9 eV together with a smaller shoulder near 5.1–5.2 eV. Because this sample lacks any aromatic substituents, its π features arise almost entirely from the CNT itself.^27^ The main π-band peak at approximately 4.9 eV remains relatively intense, and is in fact slightly stronger than the analogous peak in ACC–CNT, indicating that the π electrons of the CNT are largely unperturbed by nonaromatic surface termination. A second π feature at approximately 5.1 eV is also visible and is assigned to an additional graphitic π-state contribution, possibly associated with less-packed or edge-state regions of the CNT network.^27^ The appearance of two closely spaced π features at approximately 4.9 and 5.1 eV likely reflects the inherent valence-band structure of the CNT, including a primary π-band peak and a shoulder associated with π–π interactions, which are more clearly resolved here because the surface is not masked by additional aromatic groups.^27^ Overall, the low-binding-energy region of PDC–CNT indicates that its aromatic character is derived solely from the CNT backbone, while the long sp^3^-rich alkyl chains do not contribute significantly in this energy range.^27^

ACC–CNT exhibits a broadened π region with multiple resolved features at approximately 4.9, 5.3, 5.5, and 5.8 eV. This increased substructure indicates a stronger perturbation of the CNT valence band than is observed for the other functionalized samples and is consistent with the presence of oxygen-containing groups on the linker.^27^ The feature near 4.9 eV is assigned to aromatic CNT π states, whereas the signal near 5.2–5.3 eV corresponds to the higher-binding-energy shoulder of the CNT π band.^27^ In ACC–CNT, both of these features are shifted slightly to higher binding energy, consistent with electron withdrawal from the CNT π system.^27^ The most distinctive aspects of the ACC–CNT spectrum are the additional features near 5.5 and 5.8 eV. These are consistent with oxygen-influenced occupied states introduced by the carbonyl- and hydroxyl-containing linker.^27^ The feature near 5.5 eV is reasonably assigned to a bonding state associated with the carbonyl-containing functionality, while the feature near 5.8 eV likely reflects stronger mixing between oxygen-derived orbitals and adjacent CNT states.^27^ Because UPS probes only occupied states, these features are interpreted as oxygen-perturbed valence-band contributions rather than antibonding π* states.^27–29^ Taken together, the higher binding energies and increased splitting observed in the π region indicate that the carboxyl-containing linker perturbs the CNT electronic structure more strongly than either the aromatic or aliphatic modifications.^27^ A weak shoulder at still higher binding energy provides additional support for oxygen-derived contributions to the valence band.^27^ Overall, the ACC–CNT spectrum is consistent with substantial electronic interaction between the oxygen-containing surface functionality and the CNT π framework.^27^

In the high-binding-energy σ region (7–20 eV), shown in Figure 9c, all samples display a broad hump corresponding to σ-bonded electrons associated with C–C σ bonds in the nanotube and attached functional groups, as well as C–O and C–N bonds where present. The intensity and shape of this σ band correlate with the relative amount of sp^3^ carbon and heteroatom content in each sample.^27–29^

PDC–CNT exhibits the largest σ-band intensity, with a broad valence hump extending from approximately 9 to 16 eV and reaching a maximum near 13–14 eV. This strong response is expected because PDC–CNT contains long saturated hydrocarbon chains. The numerous C–C σ bonds in these alkyl segments contribute substantially to the density of states in the 8–12 eV range.^27^ In effect, the spectrum of PDC–CNT in this region resembles that of a hydrocarbon-rich matrix superimposed on the intrinsic σ bands of the CNT. The greater intensity of the σ hump in PDC–CNT, relative to both 5PC–CNT and ACC–CNT, indicates that this sample contains the highest fraction of sp^3^ carbon, consistent with its long aliphatic chain and absence of competing aromatic substituents.^27^ PDC–CNT also exhibits a pronounced shoulder extending to approximately 16 eV, which is attributed to deeper σ states from the hydrocarbon chain and possibly the amide C–N bond. The stronger intensity of this shoulder relative to the other samples is consistent with the contribution of its extended methylene segment.^27^

5PC–CNT shows a broad σ band extending from approximately 9 to 15 eV, although its overall intensity is lower than that of PDC–CNT. This is consistent with the shorter aliphatic segment in the 5PC linker and the presence of a phenyl group, which contributes more strongly to π character than to σ intensity in this energy range.^27^ As a result, the σ envelope of 5PC–CNT is smaller than that of PDC–CNT but remains more pronounced than that of ACC–CNT, indicating that the surface functionality still contributes a meaningful aliphatic component. The σ-band maximum appears near 14–15 eV, and the overall line shape suggests mixed aromatic–aliphatic character.^27^ Thus, the valence spectrum of 5PC–CNT is consistent with the expected linker structure, namely an aromatic terminus attached through a short alkyl spacer. Overall, the spectrum combines a strong aromatic contribution with a moderate aliphatic σ background, distinguishing it from the more strongly aliphatic PDC–CNT and the more oxygen-perturbed ACC–CNT.^27^

ACC–CNT has the weakest σ-band intensity of the three samples. Only a shallow hump is present in the 10–16 eV range, indicating a much lower density of sp^3^ C–C bonds. This is consistent with the structure of the ACC functional group, which contains only a short linker and includes polar O–C=O functionalities, resulting in fewer saturated carbon atoms overall. The CNT framework remains largely sp^2^-hybridized and therefore contributes relatively little to the σ density of states when compared with a long alkyl-functionalized sample. Consequently, the σ region of ACC–CNT is comparatively flat and low in intensity. Oxygen-related σ features are nevertheless evident, as ACC–CNT displays distinct shoulders in the 13–17 eV range that are not as prominent in the other samples. These features correspond to σ electrons associated with both C–O single bonds and C=O double bonds.^27^ In particular, a shoulder near 11–13 eV can be attributed to σ orbitals of carbonyl and carboxyl functionalities unique to ACC–CNT.^27^ The presence of these features is consistent with the chemical structure of the ACC linker and provides further spectral evidence for oxygen-containing surface functionality. A minor contribution from the amide C–N bond may also be present near 9–11 eV, although this region overlaps with the tail of the broader σ band and appears to be less significant than the oxygen-related contributions.

In summary, the UPS analysis indicates that each surface modification imparts a distinct fingerprint on the valence electronic structure of the CNTs. The 5PC–CNT sample is characterized by enhanced aromatic π states arising from the phenyl group and by a moderately strong σ background associated with its short alkyl spacer. The PDC–CNT sample, which lacks aromatic substituents, emphasizes the intrinsic CNT π band while displaying a pronounced σ band arising from its long sp^3^-rich alkyl chains, consistent with the highest aliphatic content. The ACC–CNT sample exhibits multiple π features shifted to higher binding energy, indicating the influence of carbonyl- and carboxyl-containing groups, and it shows the weakest σ-band intensity because of its relatively low sp^3^ carbon content, while also displaying distinctive C–O-related features.^27^ Collectively, these UPS results provide insight into the balance between aromaticity and aliphatic character, as well as the electronic effects of oxygen-containing functional groups on the CNT surface. The peak assignments were cross-checked directly against the spectra shown in Figure 8 to ensure that the cited binding energies correspond to the plotted features for ACC, PDC, and 5PC.

## UPS Conclusions–Summary of Key Peaks and Assignments

Table 6 summarizes the key UPS peak positions observed for each sample and interprets them in terms of the CNT π-system and the contributions of the attached functional groups. All peak positions are reported in binding energy (eV), and intensities are described qualitatively. This consolidated summary highlights how the phenyl, alkyl, and carboxyl functional groups are manifested in the UPS spectra and how each modification perturbs the native valence-band features of the CNTs.^27–29^

**Table 6:** UPS Summary

sample (Curve)	observed valence peaks (binding energy)	interpretation of UPS features
**ACC**–**CNT green dotted curve**	π peaks at ∼4.9, 5.3, 5.5, 5.8 eV (broadened π-band with four subpeaks); Very small σ hump ∼10–16 eV	CNT aromatic π states appear at ∼4.9 eV and ∼5.3 eV (graphitic sp^2^ network). Higher-BE π peaksat ∼5.5 and ∼5.8 eV are due to the carbonyl/carboxyl functionalization–the C=O groups conjugate with the CNT π-system, shifting bonding π orbitals to deeper binding energy (no π* levels are detected, only occupied bonding states). The ∼5.8 eV shoulder is assigned to an O-rich π–σ hybrid orbital (involving O 2p lone-pair electrons and neighboring C π orbitals). The very weak broad feature from ∼10–16 eV indicates minimal σ-state intensity, consistent with few sp^3^ C–C bonds added. Instead, small shoulders at ∼11–13 eV in ACC–CNT’s spectrum correspond to σ electrons in C–O bonds (amide linkages and terminal −COOH), indicating the presence of oxygen functional groups. Overall, ACC–CNT’s UPS features reflect an oxygen-functionalized, partially sp^3^-disrupted CNT with multiple π-band components and low σ density
**PDC**–**CNT orange dashed curve**	π peak at ∼4.9 eV (strong); π shoulder at ∼5.1 eV; Large σ band ∼9–16 eV (highest intensity)	Graphitic π-band features dominate the low-BE region: the main CNT π peak at ∼4.9 eV is clearly observed (and is more intense in PDC–CNT than in ACC–CNT, since PDC’s surface is not covered by aromatics). A secondary π feature around ∼5.1–5.2 eV (shoulder) is also present, corresponding to the extended π states of the CNT (similar in energy to the second π peak of ACC–CNT, ∼5.3 eV). The absence of any additional aromatic groups means all π signals stem from the CNT’s intrinsic π orbitals. In the high-BE range, PDC–CNT shows the most intense σ-band of all samples, spanning ∼9–16 eV. This reflects its long aliphatic chain attachment (rich in sp^3^ C–C bonds) which contributes a high density of σ states. The broad hump peaking around ∼13 eV is characteristic of saturated carbon valence orbitals, effectively overlapping with the CNT’s own σ contributions. A slight extension of the σ intensity up to ∼16 eV indicates contributions from the pure hydrocarbon chain (and possibly the C–N amide bond) at the surface. In summary, PDC–CNT’s UPS spectrum is defined by an unaltered CNT π-band and a dominant σ feature from its long alkyl functional group, signifying the highest sp^3^ content and minimal aromatic augmentation among the samples
**5PC**–**CNT blue solid curve**	π peak at ∼4.8 eV (very intense, lowest BE); CNT π shoulder ∼5.2 eV; σ hump ∼9–15 eV (moderate intensity)	A strong aromatic π peak at ∼4.8 eV is the hallmark of 5PC–CNT, arising from the attached phenyl rings (the benzene π orbitals at the surface yield a high-intensity HOMO feature at slightly lower binding energy than the CNT’s own π peak). This is the most intense π peak among the three samples and occurs at the lowest binding energy, underscoring the enhanced aromaticity due to the phenyl functionalization. A secondary π contribution around ∼5.2 eV is visible as a shoulder, which corresponds to the CNT’s inherent π-band (present but partially overshadowed by the phenyl-derived signal). Thus, the π region of 5PC–CNT combines surface benzene π states(dominant) with underlying CNT π states (weaker). In the σ region, 5PC–CNT displays a broad hump from ∼9 to 15 eV with intermediate intensity. The magnitude of this σ band is lower than in PDC–CNT (since 5PC’s functional group is shorter and includes an aromatic ring, reducing the total number of sp^3^ bonds) but greater than in ACC–CNT. It represents the σ-electron contribution of the small alkyl linker plus the CNT’s own σ framework. The 5PC–CNT spectrum thus indicates a mixed character: substantial aromatic π-content (phenyl + CNT) and a modest amount of aliphatic σ character. No additional distinct oxygen-related features appear (consistent with the phenyl linker being hydrocarbon in nature). Overall, the UPS profile of 5PC–CNT indicates that benzene functionalization intensifies π-electron signals while only moderately increasing the σ-electron background, due to the short sp^3^ tether connecting the aromatic group to the CNT

One additional analysis derived from the UPS and XPS data is the C KLL Auger *D*-parameter. Although the full Auger spectrum is not shown here, the *D*-parameter was calculated for each sample from the XPS data. For pristine CNTs, which are predominantly sp^2^-hybridized, the *D*-parameter is typically reported to be approximately 20–21 eV.^27^ For the functionalized samples, the calculated values were approximately 18 eV for 5PC–CNT, 16.5 eV for PDC–CNT, and 17.5 eV for ACC–CNT.^27^ These values are consistent with the qualitative hybridization analysis, indicating that 5PC–CNT retains largely graphitic and aromatic character, PDC–CNT exhibits the greatest increase in sp^3^ character, and ACC–CNT shows an intermediate, mixed hybridization state. It should be noted that the present method of calculating the *D*-parameter, using KherveFitting software with a Gaussian smoothing algorithm and a smoothing width of 6, produced slightly lower values for 5PC–CNT and slightly higher values for PDC–CNT and ACC–CNT than might otherwise be expected. However, when the *D*-parameter is evaluated according to the standard definition as the energy difference between the C 1s peak and the principal Auger kinetic-energy feature, the relative ordering and approximate value ranges remain consistent with literature expectations.^27^

Overall, the UPS and *D*-parameter analyses strongly corroborate the XPS findings. The presence or absence of pronounced aromatic π-band features, together with the emergence of oxygen-related valence-band contributions in the UPS spectra, provides a distinct electronic signature for each functional group.^27–29^ In particular, 5PC–CNT retains a strong aromatic π-band and exhibits a relatively high *D*-parameter of approximately 18 eV, consistent with preservation of graphitic character. PDC–CNT shows a less prominent π-band, the most intense σ manifold, and the lowest *D*-parameter at approximately 16.5 eV, consistent with the highest aliphatic and sp^3^-rich character. ACC–CNT exhibits a broadened π region with multiple components, a comparatively weak σ contribution, and higher-binding-energy oxygen-related features, while its intermediate *D*-parameter of approximately 17–17.5 eV reflects a mixed hybridization state influenced by both sp^3^ carbon content and the electron-withdrawing character of the carboxyl-containing linker.^27^

### EDX Compositional Analysis

Although XPS and UPS provide detailed chemical-state information, EDX offers a straightforward assessment of elemental composition and complements the spectroscopic data with more bulk-sensitive quantification.^14^ EDX was therefore performed on representative samples drop-cast onto gold electrodes on Si/SiO_2_ substrates, and the resulting atomic percentages were summarized in Table 1 (representative spectra in Figure 10) and are discussed here in context.

**Figure 10:**
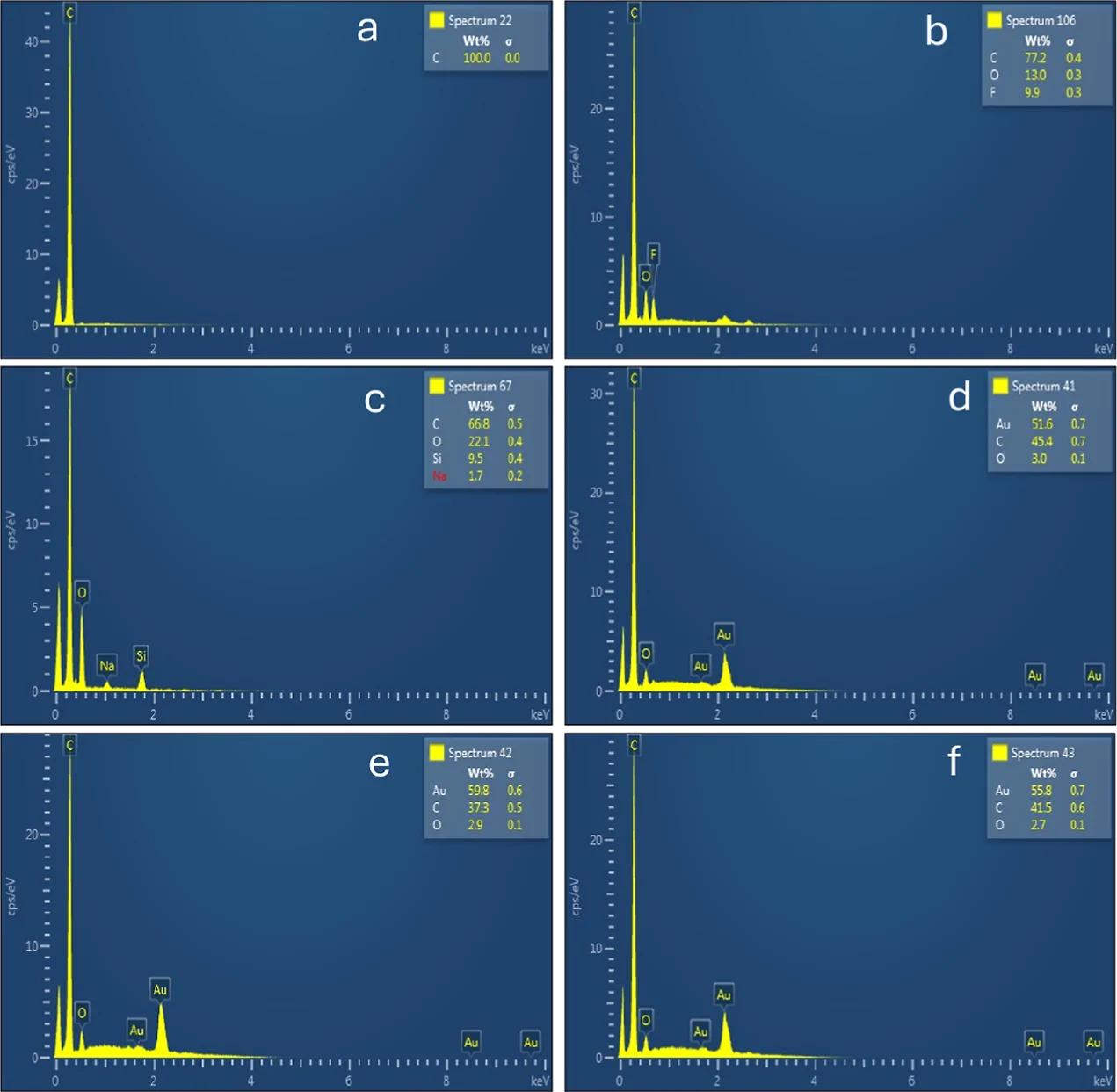
*EDX Results.* (a) nonfunctionalized CNTs, (b) OH-functionalized CNTs, (c) APTES-functionalized CNTs, (d) 5PC CNTs, (e) ACC CNTs, (f) PDC CNTs.

For unfunctionalized CNTs, EDX is expected to show carbon as the dominant element with only a small residual oxygen contribution arising from adsorbed oxygen species or minor oxidation during handling.^14^ After KMnO_4_/H_2_SO_4_ treatment, the oxidized CNTs exhibited an oxygen content of approximately 10–15%, depending on spot location and normalization, indicating substantial oxygen uptake consistent with oxidative functionalization of the CNT surface.^16,19–21^ In early measurements collected on gold substrates, an apparent fluorine peak near 0.7 keV was also observed. However, blank-substrate measurements indicated that this feature was present even in the absence of a sample, supporting the conclusion that it arose from a substrate-related artifact rather than from true fluorine in the CNT material. Overall, the increase in oxygen content is consistent with prior reports for oxidized CNTs, which commonly show substantial incorporation of oxygen-containing surface groups after similar treatments.^16,19–21^

The EDX results for APTES-functionalized CNTs were especially informative. After correction, APTES–CNT samples typically showed carbon in the range of approximately 65–75%, oxygen in the range of 15–25%, nitrogen in the range of 1–5%, and silicon in the range of 10–15%. These values are consistent with expectations for APTES-modified CNTs because APTES introduces both nitrogen- and silicon-containing functionalities, while the oxygen signal reflects contributions from both the silane layer and the pre-existing oxidized CNT surface.^22–24^ The appearance of nitrogen and silicon, which were absent in the original CNT material, therefore provides strong qualitative evidence for successful silanization.^22–24^ Although some substrate-related interference near the silicon region is possible, XPS is expected to provide more reliable quantitative information for light elements and low-abundance surface species.^14^ Even so, the EDX data clearly demonstrate the appearance of new silicon- and nitrogen-related signals after APTES treatment, which supports successful incorporation of the silane linker.

EDX measurements of the final functionalized materials showed trends consistent with the expected chemistry of the attached groups. In 5PC–CNT, the measured composition was dominated by carbon and substrate-derived gold, with only a small oxygen contribution and no detectable chlorine. The absence of chlorine is consistent with reaction of the acyl chloride with surface amines followed by removal of HCl as a byproduct, while the increased carbon contribution is consistent with attachment of a phenyl-containing linker. PDC–CNT similarly showed no detectable chlorine, again supporting successful reaction of the long-chain acyl chloride with the amine-functionalized surface. Its carbon-rich composition is consistent with the incorporation of a saturated hydrocarbon chain. ACC–CNT also showed no chlorine above the detection limit, which is consistent with covalent attachment through amide-forming chemistry. Compared with 5PC–CNT and PDC–CNT, ACC–CNT is distinguished by its relatively greater oxygen-related contribution, in agreement with the oxygen-containing nature of the carboxylated linker. Across all three samples, the strong gold signal is attributed to the substrate rather than to the CNT material itself.

Taken together, the EDX data provide three broad conclusions. First, the appearance of nitrogen- and silicon-related signals after APTES treatment supports the presence of the silane linker on the CNT surface.^22–24^ Second, when the substrate contribution is accounted for and the data are considered comparatively, ACC–CNT shows the highest relative oxygen content among the final functionalized samples, as expected for the oxygen-rich linker. Third, chlorine is not detected at significant levels in the final products, which is consistent with successful reaction and subsequent purification. Because EDX is a relatively high-throughput technique, these results also suggest that the observed elemental changes are not due merely to trace contamination but instead reflect substantial chemical modification of the CNT material.

Care must nevertheless be taken when interpreting EDX data from these samples because conductive substrates, low accelerating voltages, and overlapping spectral features can introduce errors of several atomic percent and can complicate peak assignment, particularly for light elements.^14^ The comparative trends observed here, however, namely higher oxygen content in ACC–CNT and relatively greater carbon-rich character in 5PC–CNT and PDC–CNT, remain consistent with the expected chemical differences among the functional groups. Earlier scans that showed spurious silicon- or fluorine-like features further illustrate that EDX is most useful here as a rapid screening and supporting technique rather than as the primary basis for subtle compositional discrimination. In the final interpretation, XPS and UPS provide the more reliable evidence for fine chemical-state and valence-band differences, whereas EDX serves as a useful complementary tool for qualitative detection of silicon- and nitrogen-containing functionalities and for confirming the absence of unexpected elemental impurities.^14,27^

## Summary of Analytical Findings

Taken together, the analytical results support successful stepwise functionalization of the CNT surface. The oxidation step introduced oxygen-containing functionalities, as indicated by the increase in oxygen content observed by EDX, thereby creating anchoring sites for subsequent modification.^16,19–21^ APTES treatment then introduced amine-containing silane species, as evidenced by the appearance of nitrogen and silicon in the EDX and XPS data, which is consistent with formation of an amine-terminated CNT surface.^22–24^ Subsequent reactions with 5PC, PDC, and ACC produced amide-linked surface functionalities, as indicated by the appearance of a carbonyl contribution in the XPS C 1s region and by conversion of the N 1s signal from predominantly amine to predominantly amide character.^14,23,26^ The three terminal functionalities were further distinguished by their combined spectroscopic signatures. 5PC–CNT exhibited the strongest aromatic character, including a π–π* shakeup feature in XPS and an enhanced π-band contribution in UPS, which is consistent with incorporation of a phenyl-containing group.^14,27^ PDC–CNT showed predominantly aliphatic character, with no pronounced aromatic shakeup feature, a broadened σ manifold in UPS, and the lowest *D*-parameter, all of which are consistent with the presence of a long hydrocarbon chain.^27^ ACC–CNT showed the greatest oxygenated character, including a stronger high-binding-energy carbonyl contribution in the C 1s region, subtle differences in the O 1s response, and additional oxygen-related features in UPS, which is consistent with incorporation of a carboxyl-containing linker.^27–29^ Although interpretation of any single measurement can be limited by peak overlap on a carbon-rich substrate, the combined use of high-resolution XPS, UPS, Auger analysis, and supporting elemental data provided a consistent framework for distinguishing aromatic, aliphatic, and oxygenated surface modifications.^14,27–29^ Beyond confirming the stepwise functionalization, three further findings emerge. First, core-level XPS is largely insensitive to the identity of the terminal group on a carbon-rich CNT substrate: the C 1s and O 1s envelopes of the three samples are nearly superimposable and differ mainly in the small C=O component (2.0% for PDC–CNT and 2.6% for 5PC–CNT versus 3.2% for ACC–CNT; Figure 6). Second, the UPS valence band and the C KLL D-parameter, which are sensitive to π/σ character and carbon hybridization, provide the discriminating information, distinguishing aromatic (enhanced π band at ∼4.8 eV; *D* ≈ 18 eV), aliphatic (strongest σ band; *D* ≈ 16.5 eV), and carboxyl-terminated (split, higher-binding-energy π features; *D* ≈ 17.5 eV) surfaces. Third, N 1s analysis indicates that the common APTES–amide route converts most accessible surface amines to amides for all three acyl chlorides, with the highest conversion for ACC–CNT. Together, these findings define a practical, low-sample-requirement spectroscopic workflow for verifying chemically distinct functionalizations on carbon-rich sensing materials.

## Discussion

In this study, CNTs were covalently functionalized with three distinct molecular moieties, and the resulting surface chemistry was evaluated using complementary analytical techniques. The combined data support successful progression through each functionalization step and also highlight practical considerations associated with chemical modification of carbon-rich substrates. The discussion below therefore focuses on functionalization efficiency, analytical limitations, and the implications of these findings for sensing applications.

### Efficiency of Functionalization and Degree of Coverage

The APTES silanization step appears to have been effective. Both XPS and EDX detected silicon- and nitrogen-containing species after this treatment, indicating that a substantial fraction of accessible CNT surface sites was modified with APTES.^22–24^ Under the reaction conditions used here, the observed surface composition is consistent with at least partial monolayer coverage and possibly localized multilayer or oligomerized silane domains, which is also consistent with prior reports of APTES deposition under relatively strong reaction conditions.^22,23,30^

Quantitative XPS analysis further supports substantial surface coverage. For APTES–CNT, the N:C atomic ratio was approximately 3:100, while the O:C ratio attributable to the silane layer was estimated to be approximately 5–10% after excluding pre-existing oxygen-containing groups. Because each APTES molecule contributes one nitrogen atom, these values suggest that a substantial fraction of the CNT surface within the XPS probing depth was associated with grafted APTES. This interpretation is consistent with prior XPS studies of chemically functionalized CNTs and with reports showing that APTES treatment can generate relatively dense surface coverage under sufficiently strong deposition conditions.^14,22–24^

The N 1s spectra further suggest that most surface amines were converted to amides in all three functionalized samples. This trend was most pronounced for ACC–CNT, in which only a small residual amine contribution remained. Taken together, these results support relatively high coupling efficiency across the series. The residual amine signal observed in the 5PC–CNT and PDC–CNT samples most likely reflects incomplete accessibility of a fraction of the surface amines rather than failure of the coupling chemistry itself. Nonuniform silane coverage, partial burial of amines within the APTES layer, and local steric effects may all contribute to this behavior.^22,23,30^ Even for ACC–CNT, where the adipoyl chloride intermediate was generated *in situ*, the strong amide contribution indicates that sufficient reactive species were present to convert most accessible amines. No spectroscopic evidence for extensive cross-linking beyond ordinary CNT bundling was observed.

### Structural Integrity after Oxidation

Acidic KMnO_4_ oxidation can introduce sidewall defects and cut nanotubes when used at high oxidant loadings and long reaction times.^16,19,21^ Raman spectroscopy (D/G band intensity ratio) would provide the most direct measure of defect density but was not available for this study. Several observations nevertheless indicate that the conjugated sp^2^ framework was largely retained. First, all functionalized samples exhibit a well-defined π band at ∼4.8–4.9 eV in UPS (Figure 9b), which requires extended delocalized π states. Second, the C 1s spectra of all samples remain dominated by a narrow C–C component (fwhm ≈0.86 eV; 82–84% of the C 1s area; Tables 3–5). Third, the C KLL D-parameters (16.5–18 eV) remain well above values typical of sp^3^-dominated carbon,^27^ and the decrease relative to pristine CNTs is consistent with the added sp^3^ carbon of the grafted chains. These indicators are qualitative; quantification of oxidation-induced damage by Raman spectroscopy and electron microscopy is recommended for future work.

### Spectroscopic Differentiation of Functional Groups on a Carbonaceous Background

A central challenge in this work was distinguishing surface-bound functional groups from the dominant carbon signal of the CNT framework. This difficulty is well recognized in the XPS analysis of carbon nanomaterials, where the intrinsic carbon backbone, oxygen-containing surface species, and adventitious carbon can complicate both peak assignment and quantitative interpretation.^14,27,31,32^ The present results show that this challenge can be addressed more effectively through combined analysis of high-resolution XPS and UPS data rather than through reliance on any single spectral feature.^14,27–29^

The aromatic functionality was distinguished most clearly by an increase in π character, as reflected in both the *D*-parameter and the UPS response.^27^ In contrast, the aliphatic functionality was associated with greater sp^3^-like carbon character and a reduced π contribution relative to the aromatic sample.^27^ Although these differences are subtler than those introduced by oxygen-containing functionalities, they remain distinguishable through side-by-side comparison of the functionalized CNT series. The carboxyl-containing functionality was the most readily assigned because it produced more direct oxygen-related signatures in the C 1s and O 1s regions together with corresponding features in the UPS spectra.^14,27–29^ More broadly, the use of three chemically distinct surface modifications strengthened the analysis by providing an internal comparative framework. Without such comparison, assignment of the bound functional groups would have been substantially less certain.

### Implications for Sensing Pre-Seizure Biomarkers

Sensor performance was not measured in the present study; the following discussion therefore describes expected, rather than demonstrated, sensing behavior. The primary motivation for this work was to tailor CNT surfaces toward different chemical affinities relevant to biomarker sensing. Because the three functionalizations generate distinct interfacial chemistries, they would be expected to interact differently with volatile and semivolatile analytes.^2,8,13^ In this sense, the value of the present approach lies less in maximizing the response of any single material than in creating a chemically diverse sensing set that can support differential analyte recognition.^2,8,33^

The 5PC–CNT material introduces additional aromatic character at the CNT surface. This modification would be expected to favor interactions with aromatic or relatively nonpolar analytes through enhanced π interactions and dispersive forces.^2,8^ By contrast, PDC–CNT presents a more aliphatic and hydrophobic surface environment, which should preferentially interact with nonpolar analytes while reducing sensitivity to strongly polar interferents.^8,13^ ACC–CNT, in turn, introduces carboxyl-containing functionalities that create a more polar and hydrogen-bonding interface. This functionality may therefore enhance interactions with basic or highly polar analytes, although it may also increase susceptibility to humidity and other background water.^2,8,34,35^

Taken together, these three surface chemistries provide complementary sensing environments rather than minor variations of the same CNT substrate. This distinction is important because seizure-related chemical signatures, if present, are unlikely to be captured adequately by a single surface type.^1,7^ Instead, differential responses across chemically distinct CNT sensors may provide more informative recognition patterns than the absolute response of any individual material.^1,8,33^

An array composed of these functionalized CNTs, potentially alongside an oxidized CNT control, could therefore support a cross-reactive sensing strategy.^2,8,33,34^ In such an approach, each material would contribute a distinct response profile to a complex analyte mixture, and the combined response pattern could be analyzed using multivariate or machine-learning methods.^2,8,34,36^ The broader implication of the present functionalization strategy is therefore not only altered analyte affinity, but also improved potential for comparative sensing across a chemically diverse sensor array.

### Overlapping Signals and Selectivity

Although the surface analyses demonstrate clear chemical differentiation among the functionalized CNT samples, all three materials remain carbon-based conductors that share a common transduction mechanism. During sensor operation, the measured electrical response would still be expected to arise primarily from changes in CNT network conductivity induced by analyte adsorption.^8,12,13^ The key question, therefore, is not whether the readout mechanism changes, but whether the surface functional groups alter adsorption sufficiently to generate distinct response behavior.^2,8,13^

The present results are consistent with that expectation, although it remains to be tested by device measurements. The underlying CNT network provides the electrical pathway, whereas the grafted surface groups are expected to modulate which analytes adsorb and how strongly they interact with the modified interface.^2,8^ As a result, selectivity would be expected to arise from differences in interfacial chemistry rather than from changes in the fundamental conduction mechanism.^8,12,13^ This distinction is important for interpretation of future sensing data because differential responses across the functionalized CNT series would most likely reflect differences in adsorption and local electronic perturbation at the modified surface rather than changes in the intrinsic transport mechanism of the CNT network.^2,8,33^

### Challenges and Future Work

A remaining challenge is the inhomogeneity of functionalization across the CNT network. The EDX results suggest that some regions retained more residue than others, which is consistent with incomplete dispersion, uneven reaction access, or incomplete washing. CNT bundling likely contributes to this behavior by limiting access to internal surfaces and making some nanotubes less available for functionalization than others.^15,16^ The small residual π feature observed in UPS for the PDC–CNT and ACC–CNT samples is also consistent with incomplete or nonuniform surface coverage.^27^

This heterogeneity does not undermine the overall conclusion that functionalization occurred, but it does indicate that these materials are better viewed as distributions of surface states rather than as perfectly uniform products. For sensing applications, average coverage and reproducibility may ultimately be more important than complete functionalization of every individual nanotube. Future work should therefore focus on improving reaction uniformity and on quantifying functional-group loading more directly.^15,16^

Another practical challenge is signal overlap arising from substrates or from the surrounding measurement environment. As observed during EDX analysis, substrate contributions can complicate spectral interpretation if they are not carefully controlled.^14^ Similar considerations are likely to apply in device measurements, where electrode materials, substrates, and environmental background may all influence the observed response. Careful device design and baseline calibration will therefore be necessary to separate CNT-derived signals from contributions unrelated to the intended surface chemistry.^8,12^

### Distinguishing Challenges due to Carbon Background

A central challenge in this work was distinguishing signals from the grafted functional groups against the dominant carbon background of the CNT substrate. Because both the nanotube framework and much of the attached chemistry are carbon-rich, elemental contrast alone provides only limited differentiation.^14,27^ As a result, identification depended on heteroatom signals together with more subtle spectroscopic differences associated with bonding environment and hybridization.^14,27,30,31^

The combined XPS and UPS analysis helped address this problem by providing complementary signatures for each surface modification.^14,27–29^ Functional groups containing distinct heteroatoms were more readily assigned, whereas hydrocarbon-rich modifications required greater reliance on comparative spectral features. In this respect, the use of multiple characterization modes was essential. No single measurement provided complete discrimination across all three functionalizations, but the combined data set allowed the surface chemistries to be distinguished with substantially greater confidence.^14,27^

More broadly, these results highlight a general consideration in the chemical modification of carbonaceous materials: functional groups are most easily verified when they introduce a distinct spectral handle that can be differentiated from the substrate background.^14,27^ In systems for which elemental composition changes only minimally, comparative analysis and complementary spectroscopies become especially important.^27–29^

### Applications and Next Steps

With the surface chemistry established, the next step is integration of these functionalized CNTs into device platforms for electrical sensing of volatile and semivolatile biomarkers. Because the 5PC, PDC, and ACC modifications provide distinct aromatic, aliphatic, and oxygenated surface environments, they would be expected to produce differential interactions with chemically diverse analytes and therefore distinct response profiles.^2,8,33,34^ In this context, the most useful outcome may not be selective detection by a single material, but rather differential response across a modular array of chemically distinct CNT sensors.^2,8,33^

Future studies should therefore focus on correlating surface functionality with device-level behavior. This includes measuring baseline electrical properties, evaluating responses to representative analyte classes, and determining whether the spectroscopically observed differences in surface chemistry translate into reproducible sensing contrast.^8,12,13^ Response libraries generated from controlled exposures could then be used for compound discrimination or for pattern-recognition-based analysis of more complex mixtures relevant to noninvasive monitoring.^2,8,34^

The UPS results further suggest that functionalization alters the valence electronic structure of the CNT network, indicating that surface modification may influence not only analyte affinity but also baseline conductivity and transduction behavior.^8,12,27–29^ Device-level studies should therefore consider both interfacial chemical interactions and the extent to which functionalization perturbs the underlying electrical properties of the CNT material.^8,12,13^

Taken together, these results demonstrate that complementary surface-sensitive techniques can distinguish chemically distinct CNT modifications despite the strong carbon background of the substrate.^14,27–29^ More broadly, this analytical framework may be useful in the development of CNT-based sensor arrays for clinically relevant biomarkers, including ketones associated with diabetes, nitrogen-containing compounds linked to chronic kidney disease, and volatile species implicated in preictal seizure states.^1–3,5–8^

In summary, the results support successful stepwise functionalization of CNTs with aromatic, aliphatic, and oxygenated surface groups and demonstrate that these surface chemistries can be distinguished spectroscopically.^14,27^ These findings provide a foundation for continued evaluation of functionalized CNTs in sensing platforms and for testing whether distinct surface chemistries produce differential response behavior toward target biomarker classes.^2,8,33^

## Conclusion

This study describes the covalent functionalization of CNTs with three chemically distinct acyl chloride-derived surface groups, namely 5PC, PDC, and ACC, together with a surface-analysis framework for verifying and differentiating each modification. A multistep wet-chemical approach was used in which CNTs were first oxidized with KMnO_4_/H_2_SO_4_ to introduce oxygen-containing surface sites, then silanized with APTES to generate surface-bound amines, and finally coupled to the target acyl chlorides to form amide-linked aromatic, aliphatic, or oxygenated terminal functionalities. The combined analytical results support successful progression through each step of this functionalization sequence.

X-ray photoelectron spectroscopy, ultraviolet photoelectron spectroscopy, energy-dispersive X-ray spectroscopy, and C KLL Auger analysis provided complementary evidence for successful modification and enabled differentiation of the resulting CNT surfaces despite the strong carbon background of the substrate. 5PC–CNT exhibited the strongest aromatic character, as reflected by the pronounced aromatic shakeup contribution in XPS and the enhanced low-binding-energy π response in UPS. PDC–CNT showed reduced π character together with a stronger σ contribution and the lowest *D*-parameter, consistent with increased aliphatic and sp^3^-rich surface character. ACC–CNT was distinguished by stronger oxygen-related spectral contributions, including enhanced carbonyl-associated features and oxygen-influenced valence-band structure, together with an intermediate *D*-parameter consistent with mixed hybridization. Taken together, these results show that orthogonal surface-sensitive measurements can distinguish aromatic, aliphatic, and oxygenated functional groups even on a carbon-rich CNT framework.

More broadly, these findings support the central premise introduced at the outset of this work: that chemically distinct CNT surface modifications can be used to create a modular interaction palette for volatile and semivolatile biomarker sensing. Rather than optimizing a single CNT surface for one analyte class, this approach establishes a set of complementary interfaces with different expected affinities for aromatic, nonpolar, and polar or hydrogen-bonding species. In this way, the value of the present platform lies not only in successful CNT functionalization, but also in providing a chemically differentiated set of CNT surfaces on which comparative sensing can be tested. Sensor response was not measured in this study; the sensing roles proposed for 5PC–, PDC–, and ACC–CNT are hypotheses based on their surface chemistry and remain to be validated in device-level measurements.

Overall, this work establishes a practical route for preparing and verifying chemically diverse CNT interfaces and provides a foundation for future device-level studies aimed at differential sensing of clinically relevant biomarkers. Such studies will be needed to determine how the spectroscopically verified surface chemistries translate into electrical response patterns for target analyte classes relevant to noninvasive monitoring, including preictal seizure biomarkers as well as markers associated with metabolic and renal disorders.

## Data Availability

Raw XPS/UPS files are available on reasonable request.

## ∥

Ashley Galanti, AMG Detection, Inc., Peachtree Corners, GA 30092, United States

## Abbreviations

CNT: carbon nanotube
SWCNT: single-walled carbon nanotube
XPS: X-ray photoelectron spectroscopy
UPS: ultraviolet photoelectron spectroscopy
EDX: energy-dispersive X-ray spectroscopy
SEM: scanning electron microscopy
APTES: 3-aminopropyltriethoxysilane
5PC: 5-phenylvaleryl chloride
PDC: pentadecanoyl chloride
ACC: adipoyl chloride
DMF: *N*,*N*-dimethylformamide
THF: tetrahydrofuran
BE: binding energy
VOCs: volatile organic compounds.
